# Avian influenza transmission risk along live poultry trading networks in Bangladesh

**DOI:** 10.1038/s41598-021-98989-4

**Published:** 2021-10-07

**Authors:** Natalie Moyen, Md. Ahasanul Hoque, Rashed Mahmud, Mahmudul Hasan, Sudipta Sarkar, Paritosh Kumar Biswas, Hossain Mehedi, Joerg Henning, Punam Mangtani, Meerjady Sabrina Flora, Mahmudur Rahman, Nitish C. Debnath, Mohammad Giasuddin, Tony Barnett, Dirk U. Pfeiffer, Guillaume Fournié

**Affiliations:** 1 Department of Pathobiology and Population Sciences, Royal Veterinary College, University of London, Hatfield, Hertfordshire AL9 7TA UK; 2 Department of Medicine & Surgery, Faculty of Veterinary Medicine, Chattogram Veterinary and Animal Sciences University, Chattogram, 4225 Bangladesh; 3 Animal Health Research Division, Bangladesh Livestock Research Institute, Dhaka, 1341 Bangladesh; 4 Institute of Epidemiology, Disease Control and Research (IEDCR), Dhaka, Bangladesh; 5 Department of Microbiology and Veterinary Public Health and Director, Poultry Research and Training Center (PTRC), Chattogram Veterinary and Animal Sciences University, Chattogram, 4225 Bangladesh; 6 Department of Livestock Services, Dhaka, Bangladesh; 7 School of Veterinary Science, The University of Queensland, Gatton, QLD 4343 Australia; 8 Department of Infectious Disease Epidemiology, London School of Hygiene and Tropical Medicine, London, WC1E 7HT UK; 9 Additional Director General Planning and Development Directorate, General of Health Services, Dhaka, 1212 Bangladesh; 10 The Firoz Lalji Centre for Africa, London School of Economics and Political Science, London, UK; 11 Humanitarian and Conflict Response Institute, University of Manchester, Manchester, UK; 12 Jockey Club College of Veterinary Medicine and Life Sciences, City University of Hong Kong, Hong Kong, SAR China

**Keywords:** Computational models, Dynamic networks, Population dynamics

## Abstract

Live animal markets are known hotspots of zoonotic disease emergence. To mitigate those risks, we need to understand how networks shaped by trading practices influence disease spread. Yet, those practices are rarely recorded in high-risk settings. Through a large cross-sectional study, we assessed the potential impact of live poultry trading networks’ structures on avian influenza transmission dynamics in Bangladesh. Networks promoted mixing between chickens sourced from different farming systems and geographical locations, fostering co-circulation of viral strains of diverse origins in markets. Viral transmission models suggested that the observed rise in viral prevalence from farms to markets was unlikely explained by intra-market transmission alone, but substantially influenced by transmission occurring in upstream network nodes. Disease control interventions should therefore alter the entire network structures. However, as networks differed between chicken types and city supplied, standardised interventions are unlikely to be effective, and should be tailored to local structural characteristics.

## Introduction

Live animal markets are known to be a source of zoonotic pathogens for humans^1^. By bringing animals of different species and geographical origins into close contact, markets promote the transmission, evolution, and, possibly, the zoonotic transfer of pathogens carried by marketed animals. In order to control the spread of zoonotic diseases’ through these, attempts have been made to ban live animal markets. Such bans have targeted the marketing of live birds in response to outbreaks of avian influenza A(H5N1) and A(H7N9)^2^, as well as the marketing of wild animals during the SARS^1^ and the 2013–2016 West African Ebola^3^ epidemics. The possible role played by a wildlife market in the initial spread of SARS-CoV-2 led to the prohibition of wild animal marketing across China; and voices have called for this prohibition to be extended globally^4^. However, indiscriminate ban of live animal markets, wild or domestic, is difficult to enforce. Ignoring the trading networks in which those markets are embedded may have unintended social and epidemiological consequences. Firstly, it may adversely affect vulnerable populations whose livelihoods heavily rely on transactions operated through those networks^5^. Secondly, failing to account for the continuing demand and network actors’ motivations may not eliminate, but alter those networks, driving them underground, thus promoting pathogen dissemination^6^, ultimately making it even more difficult to monitor and regulate them^3^.

As an alternative to market bans, a range of technical interventions—cleaning and disinfection, ban on overnight poultry storage, usage of personal protective equipment—have aimed at reducing pathogen burden and human exposure in markets, while minimising trade disruption. Although these measures have been effective in Hong Kong^7^, in other settings the lack of adaptation of those measures to local contexts meant that they had limited effectiveness^8–11^. The way in which markets are connected within live bird trading networks is generally ignored, as the networks’ configurations are often undocumented. Yet, in order to mitigate zoonotic and animal disease risks associated with live animal trade more effectively, as well as to detect the emergence of pathogens of public health importance more rapidly, measures need to be based on an understanding of how those networks are structured, and how their configurations influence disease risks.

Since its emergence in 2007, highly pathogenic avian influenza (HPAI) A virus subtype H5N1 has been circulating endemically in Bangladesh along with multiple other avian influenza virus (AIV) subtypes^12^. Those viruses are ubiquitous in live bird markets^13^, through which nearly all poultry consumed in Bangladesh transit^14^. In the absence of systematic recording of live bird traders’ movements, labour-intensive data collection is needed to characterise the networks in which markets are embedded. This usually limits study scale, and, while such studies have been useful to identify epidemiologically relevant features of those networks, they could not unravel their overall structure^15,16^. To address this gap, we conducted a large-scale study aimed at assessing the configuration of the live poultry trading networks supplying the two largest Bangladeshi cities, Dhaka and Chattogram. We show how detailed characterisation of the configuration of live poultry trading networks can improve our understanding of AIV transmission dynamics in an endemic area and support the development of more effective surveillance and control programmes.

## Results

**Figure 1:**
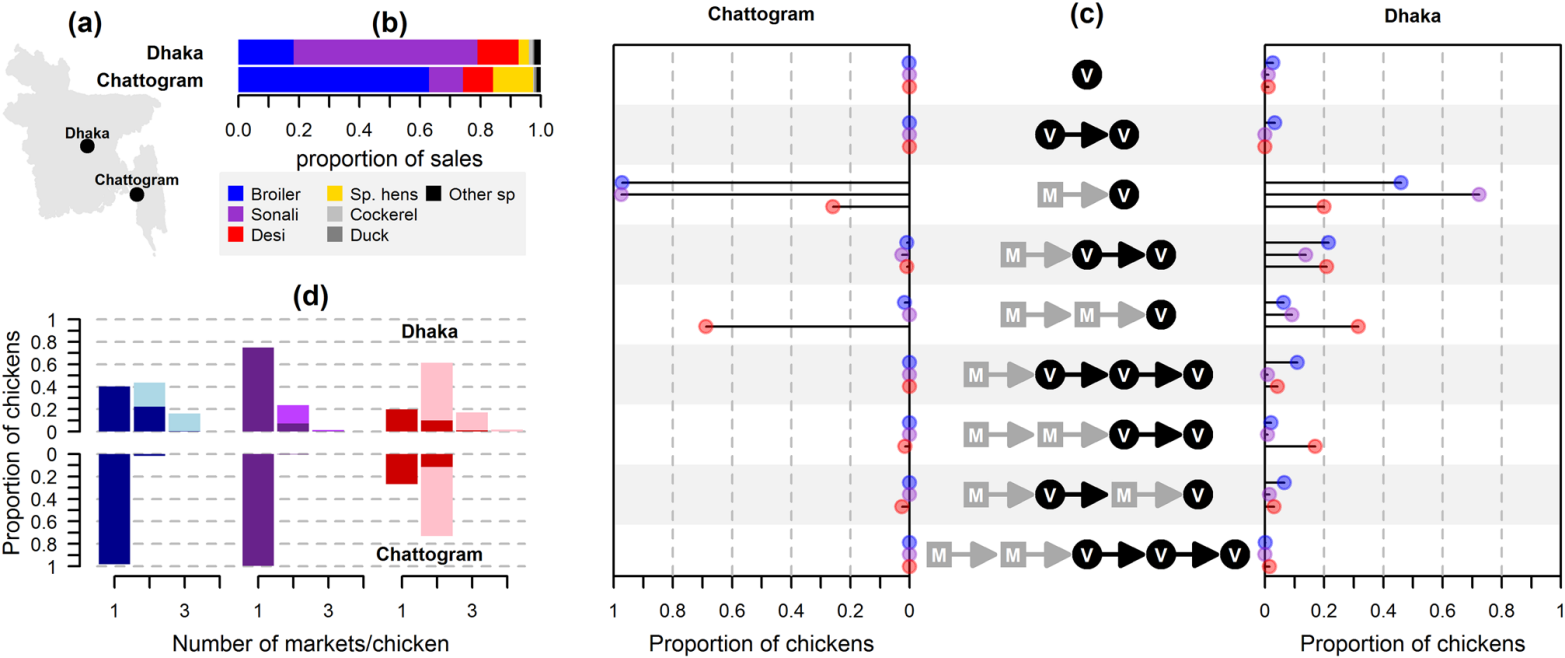
Type of poultry and chains of actors. (**a**) Map of Bangladesh; (**b**) proportion of sales of each poultry type in each city; Sp. hens: spent hens, Other sp: other species; (**c**) proportion of chickens traded through each possible combination of actors, supplying both cities in broilers (blue), sonalis (purple) and deshis (red); *M* mobile trader, *V* market vendor; (**d**) proportion of broilers (blue), sonalis (purple) and deshis (red) sold in Dhaka and Chattogram according to the number of markets through which they transited; darker: only involving markets in the supplied city, lighter: involving at least one market located outside the supplied city. Catchment areas were mapped using maptools^57^, road distances were computed using gmapsdistance^58^.

In the following, live bird markets are referred to as markets. Through a cross-sectional study, conducted from March to September 2015, we interviewed 1389 *vendors* holding a permanent stall in one of 119 markets visited in Chattogram and Dhaka (Fig. 1a), and 520 *mobile traders* supplying live birds to those vendors, either from other markets or farms (sometimes referred to as *middlemen*). They were asked about their trading practices in the 7 days preceding the interview. In addition, a cohort of 43 vendors and 37 mobile traders was interviewed monthly, in order to assess how their practices varied over a year.

### Types of poultry sold in markets

We estimated that more than 97% of poultry transiting through Dhaka and Chattogram’s market stalls were chickens, and less than 0.7% were ducks (Fig. 1b, Tables S3–S7). Other species included geese, quail and pigeons. Chickens were described by network actors according to their *type*, based on their perceived breed, and production purpose. Overall, *broiler*, *sonali* breeds and *deshi* chickens accounted for 95.1% (2.5–97.5th quantile range, 95% R: 94.8–95.6%) and 86.2% (95% R: 85.8–86.5%) of chickens sold in Dhaka and Chattogram, respectively, and *spent hens* and *cockerels* for the remainder. The most frequently traded chicken types were sonalis in Dhaka and broilers in Chattogram (Fig. 1b). Broilers referred to exotic broiler, fast-growing chicken breeds (e.g. Ross, Cobb 500)^14^. Deshis were indigenous, often non-descript, chicken breeds^17^. Phenotypically close to deshis, sonalis were a crossbreed (F1 generation of Fayoumi (Female) and Rhode Island Red Male) introduced to Bangladesh in the 90’s by rural development programmes^18^. Sales of chickens tended to decrease during the rainy season (June–August) (Table S19).

### Sequences of actors

From here onwards, we focus on broilers, sonalis and deshis, the main poultry types traded in the study area. The sequences of actors, defined as the number of successive traders involved in the trade of chickens from farms through to markets differed according to chicken type and city supplied, with relatively shorter sequences of actors for broilers and sonalis than for deshis, and for Chattogram than for Dhaka (Fig. 1c). Likewise, the number of markets through which chickens transited was higher for deshis than for broilers and sonalis, and for Dhaka than for Chattogram (Fig. 1d). Transactions between mobile traders and broiler or sonali farmers were often mediated by another type of actor, feed dealers, especially in Chattogram (Table S21), who supplied farmers with credit and production inputs. They connected farmers and mobile traders, but did not, themselves, transport or store chickens. Deshis were often directly sourced by mobile traders from small-scale, backyard farmers. More than 95% of chickens leaving Chattogram markets (i.e. not transiting through another market stall) were sold to end-users (e.g. consumers, restaurateurs, caterers), and were therefore slaughtered at the market or soon after the end-point transaction. In contrast, in Dhaka, a quarter of broilers, a third of deshis and most sonalis were estimated to be sold to other traders operating outside markets (e.g. hawkers, street shops), and were therefore kept alive for longer periods of time (SI, Table S20).

Deshis were sold at a higher price than broilers and sonalis, but prices of all chicken types were lower in Dhaka than Chattogram, despite more actors and markets being involved (Table S16).

Almost all mobile traders (97.1%, n = 505) reported trading a single poultry type in the week preceding the interview (Table S9), whereas two-thirds of vendors (65%, n = 486) sold several types at their stall (Table S8), suggesting that mixing between poultry types remained limited until reaching market stalls.

### Market catchment areas

**Figure 2:**
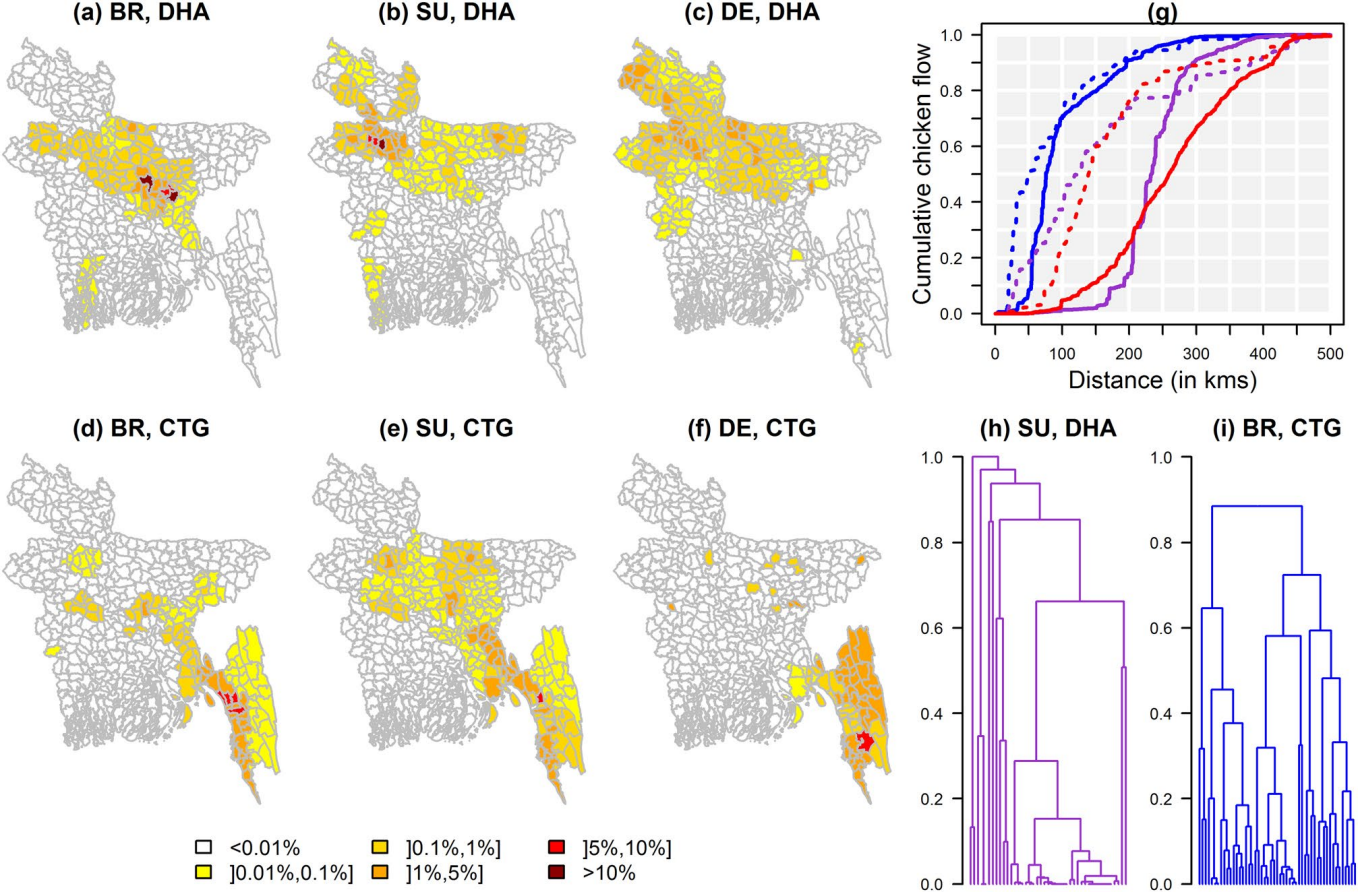
Origins of chickens sold in Dhaka and Chattogram markets. Catchment areas of Dhaka (**a–c**) and Chattogram (**d–f**) are shown for broilers (**a,d**), sonalis (**b,e**) and deshis (**c,f**); (**g**) cumulative distribution of broilers (blue), sonalis (purple) and deshis (red) sold in Dhaka (solid lines) and Chattogram (dashed lines) according to the distance over which they were transported, from farms to their terminal market; (**h,i**): hierarchical clustering of Dhaka and Chattogram markets based on the similarity of their catchment areas for sonalis and broilers, the most commonly chicken types in each city, respectively; the distance between any two markets (y-axis) was 1-[Pianka index], with a value of 0 meaning that two markets had similar catchment areas, and a value of 1 that those areas did not overlap.

We explored the networks’potential to mix chickens of different types and from different geographical origins. A catchment area describes the set of upazilas (sub-district) where farms supplying a market, or markets within a city, were located. Broilers were sourced near cities, 70% being transported, from farms to terminal markets, over < 100 km (Fig. 2g). In contrast, most chickens, 59.4–85% according to the type, estimated to originate from Chattogram district and its bordering districts. There was no, or only very limited, overlap between both cities’ catchment areas (Table 1). Dhaka’s three catchment areas (Fig. 2a–c) didn’t overlap as much as Chattogram’s (Fig. 2d–f). Three districts in the north and east of Dhaka and three in the north-west supplied 75.7% of broilers and 82.1% of sonalis, respectively. Origins of deshis were more scattered over northern and north-western districts.

**Table 1:** Pairwise comparison of catchment areas according to the city supplied and the type of chickens traded using Pianka’s overlap indices^19^.

	SO, DHA	DE, DHA	BR, CTG	SO, CTG	DE, CTG
BR, DHA	0.06 (p = 0.205)	0.15 (p = 0.076)	0.03 (p = 0.532)	0.23 (p = 0.007)	0.02 (p = 0.894)
SO, DHA		0.57 (p < 0.001)	< 0.01 (p = 0.997)	0.08 (p = 0.346)	< 0.01 (p = 1)
DE, DHA			0.01 (p = 1)	0.09 (p = 0.909)	0.01 (p = 1)
BR, CTG				0.74 (p < 0.001)	0.34 (p < 0.001)
SO, CTG					0.40 (p < 0.001)

An index of 0 meant that both catchment areas did not overlap at all, and an index of 1 that they were identical.

*BR* broiler, *SO* sonali, *DE* deshi, *DHA* Dhaka, *CTG* Chattogram; in brackets: p-value.

In order to assess potential temporal variations in these catchment areas, 37 mobile traders were asked every month, for a year, about the origins of the chickens they traded over a four-day period, referred to as the interview-period. Mobile traders purchased chickens from an average of 2 upazilas per interview-period. However, some mobile traders frequently changed the upazilas where they purchased chickens from one month to the next, resulting in them visiting a large number of upazilas (mean: 7.5–8.8, up to 17–25 depending on chicken type) and districts (average: 2.4–5.1, up to 5–14) over the study period (Table S17). These visited upazilas were nonetheless within the boundaries of the catchment areas characterised for each chicken type through the cross-sectional study.

Most markets’ catchment areas were similar, or substantially overlapped, with the catchment areas of other markets in the same city (Fig. 2h, i, SI Fig. S6). This suggested that, for each chicken type, multiple markets in a city sold chickens sourced from the same geographical areas. We then assessed the potential of chickens from different types and geographical origins to be sold together in a given market. Defining a farmed chicken population as all chickens of a given type farmed in a given upazila (or district), any two farmed chicken populations among those accounting for > 97% of catchment areas supplying each city could be sold together in at least one market (SI, Table S23) In other words, this meant that almost any two chickens supplied to Dhaka or Chattogram, regardless of their type and geographical origin could potentially be traded in the same market.

### Movements of chickens and traders between markets within cities

Although Dhaka’s and Chattogram’s markets show comparable potential for mixing chickens from different farming systems and geographical origins, transaction patterns differed greatly between both cities. In Dhaka, each interviewed mobile trader generally supplied chickens to only one market (60.8%), rarely more than two (12%), and 38.7% of vendors purchased chickens in another market than the one where they operated. In contrast, almost all vendors in Chattogram (98.7%) purchased all their chickens in the market where they operated, and most interviewed mobile traders (59.2%) sold chickens in at least five markets around the city. This resulted in networks of chicken and trader movements between markets in each city being mainly shaped by (i) inter-market transactions in Dhaka and (ii) movements of mobile traders delivering chickens to multiple markets in Chattogram. In Dhaka, out of the 64 markets we surveyed, 47 were involved in inter-market transactions, and connected within a unique component. The network was almost acyclic, and highly heterogeneous, with most nodes (74.5%) being sinks, supplied from an average of two other markets, and 63.9% of inter-market edges originated from the two largest Dhaka markets (Table S24). In Chattogram, only eight of the 55 surveyed markets traded chickens with another market. Accounting for all traders’ movements between markets (i.e. associated, or not, with inter-market transactions) did not affect the overall structure of the network in Dhaka, the degree (i.e. the number of other markets to which a market was connected) distribution remaining right-skewed, with the two largest markets still mediating most connections between other markets. In Chattogram, the resulting network was as large as Dhaka’s, but with four times as many edges, and a symmetrical, thinner-tailed, degree distribution. Both networks showed high clustering coefficients and short path lengths, suggestive of small-world properties (Table S25).

### Chicken and AIV dynamics in markets

**Figure 3:**
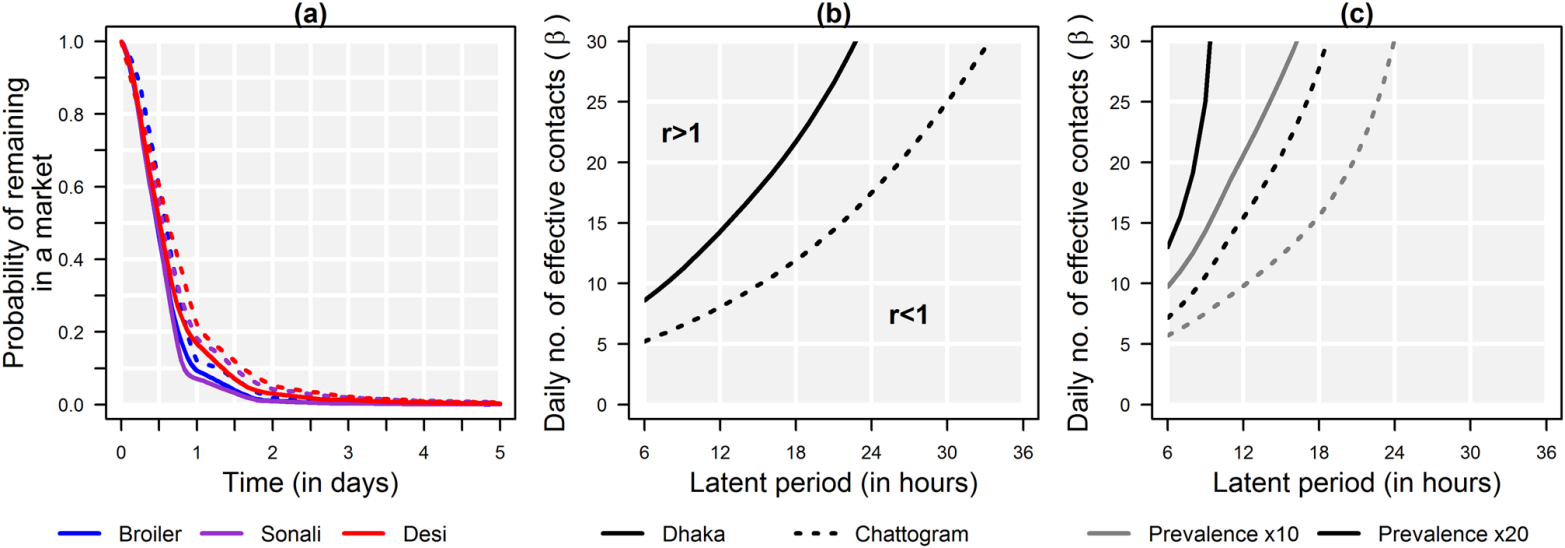
Chicken and AIV dynamics in a market. (**a**) Probability of a chicken remaining in a market as a function of time, for broilers (blue), sonalis (purple), deshis (red), in Dhaka (solid) and Chattogram (dotted); (**b**) Values of *β* required for *r* = 1 as a function of the latent period; *β* refers here to the average daily number of effective contacts (i.e. resulting in infection if involving a susceptible and an infected bird), in a closed population, accounting for both direct and environmentally-mediated transmission; values of *β* result in *r* > 1 above the solid line for Dhaka markets and dotted line for Chattogram markets, and in *r* < 1 below these; (**c**) Values of *β* required for a 10- and 20-fold increase in the prevalence of infection as a function of the latent period; grey: tenfold, back: 20-fold.

Interviewed mobile traders supplying market stalls with broilers and sonalis reported collecting them from commercial farms the night, or the day, before selling them at markets. Deshis were collected from multiple small-scale backyard flocks, over 2–3 days, to form a batch which was then dispatched to city markets. Based on trading practices reported by vendors, the length of time spent by chickens in markets was estimated to be slightly longer in Chattogram than in Dhaka, and for deshis than for other types (Fig. 3a). However, the turnover was high as the probability of a chicken remaining in a Dhaka or Chattogram market for more than 24 h was 0.09 and 0.14, respectively, and for more than 48 h was 0.01 and 0.03, respectively. This high turnover meant that for AIVs to be sustained in a marketed chicken population, i.e. reproduction number *r* ≥ 1, *β* needed to be high (Fig. 3b). *β* refers here to the average daily number of contacts resulting in infection if involving a susceptible and an infected bird, in a closed population. Depending on the city, *β* would need to reach 8.1 or 14.3, for a latent period of 12 h, and 17.5 to 32.7, for a latent period of 24 h. A 20-fold increase in the prevalence of infection for newly introduced chickens, as observed in the field with H9 AIV between farmed and marketed chickens^13,20^, could not be achieved in Dhaka markets for a latent period ≥ 12 h, due to the high turnover of chickens (Fig. 3c).

## Discussion

The latent period associated with avian influenza viral infection in poultry^21–25^ is considered to be short, lasting less than, or slightly more than a day. However, the high turnover of chickens in Dhaka’s and Chattogram’s markets meant that (i) the level of transmission required for a virus to be sustained in a marketed chicken population would need to be higher than transmission rates estimated in the literature for different subtypes and strains^21–24,26–28^, and (ii) the observed rise in prevalence from farms to markets was unlikely to be explained by intra-market transmission alone. Viral shedding in markets was therefore likely due, at least partially, to infection occurring before chickens reached markets, during their transport, and possibly their collection, from farms. Mobile traders often visit several farms to put together a shipment which they then deliver to markets or other intermediaries, using vehicles which are rarely cleaned thoroughly^11^. This is likely to promote viral transmission between birds originating from different flocks, transported within the same vehicle, at the same time or sequentially. Likelihood of transmission would further increase as multiple traders are involved. Moreover, chickens from a given shed can be sold through multiple transactions occurring sequentially over a few days^29^. Successive visits to a farm by traders who enter the shed to collect chickens may result in viral introduction among chickens present in the shed, and which will enter the trading network soon after. This suggests that, in order to reduce viral load in terminal markets, control efforts conducted there should be complemented by interventions targeting farms and mobile traders. These hypotheses, which are suggested by the network configuration and the dynamics of the chicken population transiting through it, would need to be validated by virological data, for instance, through the longitudinal sampling of groups of chickens moved from farms to terminal markets, or field experiments altering the network structure to assess the impact on viral prevalence along the network nodes.

The high diversity of AIVs circulating in Bangladeshi markets has been documented^30–33^, but the underlying factors influencing these patterns have not been explored. As mobile traders rarely traded several poultry types concurrently, there were limited opportunities for broilers, sonalis and deshis to mix along the trading networks until they reached market vendors’ stalls. Therefore, if a substantial proportion of the viral amplification observed along networks took place before chickens reached terminal markets, as hypothesized above, viruses shed by chickens at market stalls should be a sample of strains circulating in the geographical areas and farming systems where those chickens originated. Such frequent introductions of AIVs from a variety of farming systems and geographical areas may result in greater viral diversity compared to a scenario in which viral amplification only takes place in markets, leading to the persistence of a subset of strains. Almost any two chickens sourced from anywhere in a city’s catchment area had the potential to come into contact in a city’s market, suggesting that any combination of viral strains circulating in the geographical areas and farming systems supplying a city may occur in a market. Such a pattern, of which the actual occurrence needs to be further explored through virological studies and genetic analyses, could promote the co-infection of marketed poultry by genetically diverse AIVs, and, therefore, foster opportunities for viral re-assortment. Indeed, although the observed rise in prevalence from farms to markets was unlikely to be explained by intra-market viral transmission alone, viral transmission was nonetheless likely to occur within markets, especially involving the small fraction of chickens remaining in those markets for > 24 h. Segregating markets, or areas within markets, according to poultry types (associated with different farming system and geographical origin), combined with abovementioned measures aiming to reduce viral load in markets, could reduce the risk of emergence of new variants.

Although the supply of deshis involved longer sequences of actors, and longer periods of time spent at mobile traders’ collection centres where chickens are stored until the batch is large enough to be sent to markets, their prevalence of AIV infection was lower than or similar to other chicken types^13^. This might be explained by lower viral prevalence in backyard farms compared to commercial farms, or greater likelihood to have been exposed to AIVs, and to have developed some immune response, by the time they are sold^20^. Resistance of local breeds to AIVs has been suggested^34–37^, and occasionally explored^38–40^, unlike possible variations in their susceptibility and infectiousness compared with commercial breeds. Also, deshis’ transport conditions differ from those of commercial breeds. While deshis from a large number of households may mix in one shipment, those shipments—large baskets containing 100–120 chickens—are much smaller than for chickens sourced from only a few commercial farms and caged by thousands in trucks. As a result, viral transmission risk during transport may be lower for deshis than for commercial breeds. Field studies would be however required to assess the way in which different modes of transportation may impact on viral transmission.

Markets’ catchment areas greatly differed between Dhaka and Chattogram, but, within each city, they greatly overlapped, with markets also being embedded in a small-world network through the movements of their traders, with such a structure possibly promoting rapid viral dissemination^41^. Markets in the same city were therefore likely to harbour similar viral populations. Surveillance activities aiming to monitor the circulation of AIVs and to detect the emergence of new variants should target multiple cities, with complementary, non-overlapping, catchment areas, but would need to do so only in a limited number of markets in each city. As highlighted by the results of the longitudinal study, mobile traders could source their chickens from a large number of districts and upazilas. This feature may mean that they are able to seize economic opportunities in response to changes in price and availability of chickens, which may be triggered, for instance by natural disasters (e.g. floods), disease outbreaks, or religious festivals. Further work would be required to assess the impact of those constantly changing individual trading patterns on the overall network structure. This may not change the overall shape of a catchment area associated with a particular city—indeed the catchment area is likely to be constrained by locations of production units, competing markets (from other cities) and the balance between transport costs and sales prices often resulting in small profit margins—but it may promote mixing of chickens, and therefore, of their viruses.

The trading network structure creates conditions for high viral load and diversity in markets, generating zoonotic disease risk. Indeed, no, or limited, adherence to cleaning and disinfection programmes, limited availability of clean water, use of defeathering machine aerosolizing viruses contribute to the persistence of AIVs shed by poultry in the markets’ environment and exposure of humans visiting markets to these viruses^8^. Exposure is likely to be the highest for market workers who were observed to operate without any personal protective equipment, despite slaughtering large number of poultry per day and being in direct contact with manure and waste^8,42^. Therefore, characterising live animal trading networks, in which these markets are embedded, can improve our understanding of their role in generating zoonotic disease risk and our ability to identify the most appropriate targets for risk mitigation interventions. This approach implies a need for technical interventions aiming to re-wire network structures or alter the potential of a network connection to transmit and amplify infectious agents. However, to be successful, the design of these interventions should consider the social and economic processes influencing actors’ behaviours and resulting networks’ structures. This will require conducting ethnographic research that aims to understand the “market architecture” by examining social, economic and power relations within and between network nodes, rather than assuming that these networks are organised in keeping with conventional market models or according to “traditional” modes of exchange^43^. Here we discuss some hypotheses which may explain the observed patterns, suggesting the need for further studies.

The greater proportion of sonali sales in Dhaka suggests a stronger consumer preference for higher valued and more expensive poultry types in comparison to Chattogram where the relatively lower priced broilers make up a higher proportion of sales. Dhaka is the country’s primary destination for internal migration with people drawn to the city seeking higher incomes and year-round employment opportunities absent from other, more rural, areas^44^. This difference may be linked to changing consumer preferences that accompany higher incomes, education and urbanisation levels, mirroring observations on demand for rice, with the consumption of fine-grain varieties increasing with higher per capita income and education levels in Bangladesh^45^. Distinct and different forms of urbanisation, characteristic of each of the two cities, may also have influenced observed trading patterns within each city—i.e. the role of wholesalers and inter-market transactions in Dhaka in contrast to mobile traders delivering chickens directly to multiple markets in Chattogram. Classified as a mega-city, Dhaka is not a single entity, but rather an agglomeration of several very large urban settlements^46^, exhibiting a hierarchy of urbanity which is more complex than in Chattogram where social, geographical, industrial and commercial bases are markedly different. Beyond the differences in size and traffic congestion which may make it more difficult for poultry-carrying vehicles to move across Dhaka and partially explain the limited number of markets visited by mobile traders in this city, this may result in different dynamics with respect to inter-market competition, possibly affecting mobile traders’ trading patterns and prices. Variations in trading patterns and prices in both cities may also result from differences affecting upstream nodes in the respective networks. For instance, the North and North-Western rural populations supplying Dhaka with deshis were poorer and likely selling their chickens at a lower price, compared to populations supplying Chattogram^47^. These may also reflect different types of exchange relations between actors. As shown with rice marketing for which the dominance and value extraction exerted by merchants upon producers varied considerably across Bangladesh and shaped different circuits of trade^43^, exchange relations are likely to differ across the country, impacting the network structure. Of particular interest are the interlocked transactions in which farmers are often engaged with feed dealers, with the latter facilitating the sales of their chickens to mobile traders, and, by connecting farmers and mobile traders, literally shaping the network. Finally, the spatial organisation of production is also likely to influence the observed patterns. Indeed, the scale and spatial distribution of production units meant that collecting enough deshis, which are raised in backyard farms, requires visiting large numbers of households spread over large rural areas far away from the consumption centres. This is more labour intensive than collecting broilers raised in large flocks located in the peri-urban vicinity of cities and may explain the longer sequences of actors associated with the trade of deshis. While we mentioned above that the higher proportion of sales of sonalis in Dhaka likely resulted from consumer preference, it may also be influenced by the location of production units, which are highly clustered in North-western districts where this breed was initially introduced by development programmes, i.e. much closer to Dhaka than Chattogram^18,48,49^.

This study had several limitations. In the absence of registries, data collection relied on traders’ recall of their practices in the 7 days preceding the interview. This may have led to under-reporting of infrequent, occasional practices and suppliers. As we did not interview all mobile traders supplying Dhaka and Chattogram, the connectivity of the networks of markets created by traders’ movements in each city may be, therefore, even higher than estimated here. In order to reconstruct the catchment areas of Dhaka and Chattogram markets, and the sequences of actors involved in their supply, practices of non-interviewed mobile traders were imputed based on those reported by interviewed mobile traders operating in similar markets. Imputations will have had most impact on sequences of actors and catchment areas of broilers sold in Dhaka and deshis sold in Chattogram as non-interviewed mobile traders supplied around a quarter of those chickens (Table S21). The accuracy of these imputations depended on the representativeness of our sample of mobile traders. Although the large number of interviewed traders and the similarity of their practices suggested that the main features of the trading networks were captured, we could not assess whether the refusal rate resulted in a selection bias (Supplementary Information, Sect. 1.1.1). Mobile traders and vendors operating in markets outside of Chattogram and Dhaka were not interviewed, except for some deshi traders involved in supplying Chattogram. Based on field observations it was hypothesised, that such vendors were supplied by mobile traders in their markets. These mobile traders were then assumed to have sourced chickens in upazilas and districts around those markets. The estimated lower number of mobile traders included in sequences of actors supplying deshis to Dhaka compared with Chattogram may reflect the fact that some deshi mobile traders operating outside of Chattogram were recruited, contrary to Dhaka, where the length of the sequence of actors may have, therefore, been underestimated.

Several factors may have affected the reliability of our estimations of markets’ catchment areas, including the possible biases in the selection of mobile traders mentioned above. Not having interviewed all mobile traders supplying the study area, some upazilas supplying Dhaka and Chattogram may not have been identified. We could not account for the spatial heterogeneity in feed dealers’ practices when estimating the geographical origins of chickens. Nonetheless, the catchment areas presented here were consistent with those presented in a previous study^50^. In addition, the location of the main areas supplying broilers, sonalis and deshis to both cities^49,51,52^ correspond to areas where farmed chicken populations are known to be large, suggesting that our estimates are reliable. The catchment areas estimated in this and a previous study^50^ were, however, static, quantifying their temporal variation, and the factors affecting those, would be important to ensure optimal adaptation of surveillance and control programmes. Due to available data, this study focused on the three main poultry types sold in both cities. However, other poultry species (e.g. geese, ducks, quails) sold less frequently and for a higher price, may remain in markets for longer periods than chickens, and could therefore play a key role in viral reassortment. Future studies should aim to characterise practices associated with the trade of other poultry species and chicken types. Finally, this study focused on live bird markets, through which almost all poultry and poultry products are traded, but both cities counted numerous additional stand-alone live poultry shops, door-to-door vendors and a small but growing number of supermarkets selling chilled or frozen chickens. As the market share of the latter increases, poultry trading networks may change dramatically, and this will affect the pattern of viral transmission and evolutionary dynamics.

In conclusion, trading networks differed according to chicken types and cities studied here, but showed similar potential for mixing of chickens, and consequently their viruses, sourced from different farming systems and geographical areas. Viral amplification along the networks was likely fostered by transmission occurring before poultry reached markets in Dhaka and Chattogram. While these results suggest the need for interventions involving improved surveillance and control of AIVs and other zoonotic diseases, they will need to be validated through virological studies, and complemented by ethnographic investigations examining the factors shaping the observed diverse network structures which would need to be considered for risk mitigation attempts to be effective and sustainable.

## Methods

### Data collection

The cross-sectional study was conducted from March to December 2015. Fifty-five and 123 LBMs, as defined in Ref.^53^, were identified through snowball sampling (Supplementary Information, Sect. 1.1.1) in Chattogram and Dhaka, respectively. All vendors operating in identified markets in Chattogram (n = 55) were recruited (n = 461). Given the larger number of markets in Dhaka (n = 123), 64 were selected through stratified sampling, based on markets’ size and locations. In Dhaka’s markets, vendors were randomly selected, with sample size depending on markets’ size (n = 928) (Supplementary Information, Sect. 1.1.1). Interviewed vendors provided contact details of mobile traders supplying them, who were then recruited. Additional mobile traders were opportunistically recruited in the six Dhaka markets where vendors most frequently reported purchasing poultry from mobile traders. As a result, 340 mobile vendors were interviewed in Dhaka and 191 in Chattogram. Most mobile traders did not know the locations of the farms from which they bought poultry when the transactions were mediated by feed dealers. Typically, a mobile trader sent workers to a feed dealer’s shop, where they were directed to a farm from which they collected poultry. Those workers were often employed on a daily basis and could not be reliably identified. Instead, feed dealers’ contact details were provided by mobile traders, and 80 were purposely selected and interviewed (Supplementary Information, Sect. 1.1.2). Structured questionnaires were designed for vendors, mobile traders and feed dealers to collect information about trading practices, for each poultry type, in the past 7 days: the numbers of traded poultry, their origins and destinations, buying and selling frequency, surplus management and prices. An additional cross-sectional study, which exclusively investigated deshi and duck trading patterns, which were not fully captured by the aforementioned cross-sectional study, was carried out from November 2017 to March 2018, involving 21 vendors and 39 mobile traders supplying deshis and/or ducks to Chattogram markets (Supplementary Information, Sect. 1.1.3). Overall, 520 mobile traders transporting broilers, sonalis and/or deshis were recruited. Finally, in order to investigate temporal variation in trading patterns, a longitudinal study was carried out from September 2016 to August 2017. Every month, participants were asked, twice a month, about the numbers, origins, destinations and prices of poultry they traded over the past 2 days. A total of 43 vendors and 37 mobile traders and 19 feed dealers were recruited, covering the different poultry types and both cities’ market networks (Supplementary Information, Sect. 1.1.4).

### Ethical approval

Informed consent was given by each participant prior to the interview. The studies were approved by the Royal Veterinary College Ethics and Welfare Committee and the Chattogram Veterinary and Animal Science University Ethics Committee and all research was performed in accordance to relevant guidelines and regulations.

### Data analysis

#### Data management

Questionnaire data were double entered by two people using EpiData 3.1. EpiData’s double-entry and validation functions were used to identify, and correct inconsistencies. Data was then imported into R 3.5.1^54^ for analysis.

#### Reconstruction of the network of transactions

The numbers of poultry traded weekly between any two actors (i.e. farmers, vendors, mobile traders, feed dealers, end-users) were estimated based on the interview data. The algorithm is detailed in SI (Sect. 1.2.2). In brief, for each poultry type, we first estimated the number of poultry that vendors reported selling to end-users, and then reconstructed the possible chains of transactions up to the farms. In order to ensure that any poultry could not transit multiple times through a given actor, the network was acyclic. Interviewed vendors and mobile traders were categorised according to whom they purchased from and sold poultry to, such that there were no transactions between actors of the same category, and transactions between two categories were directed and not reciprocal. Missing information was addressed by imputations, detailed in SI (Sect. 1.2.2). In summary, practices of non-interviewed vendors in non-surveyed or surveyed Dhaka markets were imputed based on practices of interviewed vendors operating in the same or a *similar* market (i.e. same size and location). Likewise, in markets (in Chattogram and Dhaka) where actors reported trading poultry with mobile traders, but where no mobile traders were interviewed, practices of those mobile traders were imputed based on practices of interviewed mobile traders operating in *similar* markets. When poultry were sourced through feed dealers, the number of poultry purchased from farms in a given upazila was computed based on the upazila’s location with respect to the location of the feed dealer’s shop. Based on trading patterns reported through the cross-sectional and longitudinal surveys (broilers and sonalis), and the additional study focused on deshi and duck trade (deshis), we estimated, out of all poultry farms for which feed dealers facilitated transactions, the proportion which were located in (i) the same upazila as their store, (ii) the same district, (iii) a neighbouring district (Supplementary Information, Sect. 2.3). Based on field visits made to markets outside the study area and the opportunistic, unstructured interviews of actors, we assumed that vendors operating in markets outside the study area (and therefore not recruited) purchased chickens from mobile traders in the same markets as theirs. The origins of the chickens supplied by those mobile traders were simulated as described above for feed dealers, using the market’s upazila as the reference location. The impact of those imputations on the resulting network configurations were assessed (Supplementary Information, Sects. 2.2, 2.5).

#### Sequences of actors and chicken population dynamics

For each chicken type and city supplied, the frequency of chains of actors was computed by simulating 100,000 trajectories using the following algorithm. First, an upazila was randomly selected with a probability proportional to the number of chickens it supplied to the network. An actor was then randomly selected with a probability proportional to the number of chickens they sourced from that upazila. A possible chain of downstream actors through which a chicken transited until reaching a customer (i.e. an end-user or a trader operating outside a market) was thus constructed. For each sequence of actors, we estimated (i) the distances over which a chicken was moved, and (ii) the time spent by chickens in vendors’ flocks in each market of the study area through which it transited. The road distance between any two consecutive sites on a chain was computed using upazilas’ centroids and Chattogram and Dhaka centroids. For any movement between markets in Dhaka or in Chattogram, a standard distance of 15 km was considered. The duration that a chicken spent with vendors in a market was computed by simulating its purchase and sale times, based on practices reported by vendors such as frequency and timing of supply, storage of poultry before offering it for sale, management of poultry left unsold, similarly to Ref.^55^. The algorithm is detailed in SI (Sects. 1.2.2, 1.2.3).

#### Catchment areas

In order to assess if the level of overlap between two catchment areas, computed with Pianka’s^19^ niche overlap indices, was greater than what would be expected by chance, we used a permutation test. The p-value was the proportion of simulated indices obtained by randomly permuting upazilas which were as high as or higher than observed indices. City-level catchment areas were computed based on the reconstructed transaction networks, as mentioned above. Surveyed markets were used to impute trading practices in non-surveyed markets so, to estimate market-level catchment areas and avoid overestimating their level of overlap, we only considered markets for which the origins of the chickens offered for sale were identified through traders’ interviews. Hierarchical clustering was performed on markets, with respect to chicken type and city supplied, using $1-index$ as a measure of distance and Ward’s criteria^56^.

#### Movements of chickens and traders between markets within cities

Two networks of contacts with surveyed markets as nodes were built for each city, based on trading practices reported by interviewed actors. They were unweighted, and differed in their definition of edges: (i) a directed network with an edge being present between two markets if at least one actor purchased chickens in one market and sold these in another; (ii) an undirected network with two markets being connected if they were both visited by the same vendor or mobile trader, as such visits may result in viral spread, through the movements of infected chickens or contaminated fomites. The clustering coefficient was the frequency at which two markets connected to the same market were also connected to each other, and the average shortest path length was the average minimal number of edges required to connect any two nodes. If the clustering coefficient was higher, and the average shortest path length similar to those metrics computed for random networks (with the same number of nodes and edges), it would indicate that the empirical network showed small-world properties.

#### AIV transmission dynamics

We simulated the transmission of avian influenza viruses within a population of chickens in a market using a stochastic compartmental model implemented in discrete time, with an hourly time-step. New chickens entered daily in the market, all at the same time. Their probability of leaving the market was time-dependent, and informed by the distribution of the estimated duration that chickens spent in a market in Dhaka or Chattogram (see above). Chickens could be successively susceptible, pre-infectious, infectious, and removed or recovered—given that the transmission was density-dependent, the infection outcome did not impact the transmission dynamics. The lengths of latent and infectious periods were fixed. The model accounted for transmission through direct contacts and mediated by the environment (as detailed in Ref.^55^). The probability of a chicken becoming infected at time $t+\Delta t$ was $1-exp\left[-\tau \left({I}_{t}+\eta {C}_{t}\right)\Delta t\right]$, with $\tau$ the daily rate of transmission through direct contacts, $\eta$ the relative transmission rate from the environment, ${I}_{t}$ the number of infectious chickens and ${C}_{t}$ the environmental load at time $t$. The basic reproduction number *r* accounting for chicken dynamics in markets was computed based on the next-generation matrix (Supplementary Information, Sect. 1.2.4).

Catchment areas were mapped using maptools^57^, road distances were computed using gmapsdistance^58^, Pianka’s indices using EcosimR^59^, and network analysis was carried out using sna^60^ and igraph^61^, in R 3.5.1^54^.

## Supplementary Information

Supplementary Information.
