# Supplementary material for: Avian influenza transmission risk along live poultry trading networks in Bangladesh
Source: Sci Rep. 2021 Oct 7;11:19962. doi: 10.1038/s41598-021-98989-4 (PMC8497497; doi:10.1038/s41598-021-98989-4)
Supplement: Supplementary file 1 — Supplementary Information. [file 41598_2021_98989_MOESM1_ESM.pdf]

Title: Avian influenza transmission risk along live poultry trading networks, Bangladesh

Authors: Natalie Moyen[1*], Md. Ahasanul Hoque[2], Rashed Mahmud[2], Mahmudul Hasan[3], Sudipta Sarkar[4], Paritosh Kumar Biswas[5], Hossain Mehedi[6], Jörg Henning[7], Punam Mangtani[8], Meerjady Sabrina Flora[9], Mahmudur Rahman[10], Nitish C. Debnath[2], Mohammad Giasuddin[3], Tony Barnett[1, 11, 12], Dirk U. Pfeiffer[1, 13], Guillaume Fournié[1].

Supporting Information

Further information are provided about the data, the algorithms used to reconstruct the trading network and estimate the time spent by chickens in a market, the mathematical model of AIV transmission and parameter values. A sensitivity analysis exploring the impact of our assumptions on the reconstruction of the trading network are also presented.

Table of contents

1. Methods
    1.1. Data collection
        1.1.1. Selection of markets and vendors in Dhaka and Chattogram
        1.1.2. Selection of feed-dealers
        1.1.3. Additional investigations of deshis and ducks' trading patterns
        1.1.4. Longitudinal study
    1.2. Data analysis
        1.2.1. Longitudinal study.
        1.2.2. Reconstruction of the network of transactions.
        1.2.3. Duration that chickens spent with market vendors.
        1.2.4. AIV transmission dynamics.
        1.2.5. Mixing of farmed chicken populations in markets.
2. Results
    2.1. Types of poultry sold in markets.
    2.2. Sequences of actors
    2.3. Market catchment areas
    2.4. Mixing of farmed chicken populations in markets.
    2.5. Movements of chickens and traders between markets within cities
    2.6. AIV transmission dynamics
3. References

1. Methods

1.1. Data collection

1.1.1. Selection of markets and vendors in Dhaka and Chattogram.

A market was defined as an open space with 2 or more traders selling live poultry at least once per week and with official government authorization to do so. In the absence of a reliable sampling frame (i.e. a recently updated list of registered markets), a snowball sampling approach was used to identify all markets in the study area. The process was initiated by visiting all markets registered at Dhaka and Chattogram city corporation offices, or previously identified by the United Nations' Food and Agriculture Organisation (FAO). We aimed to (i) confirm they fitted the study definition of a market, (ii) assess the number of vendors, and (iii)

ask vendors for the names and locations of other markets they knew of. These newly identified markets were then visited following the same procedure, and so on until no new market was identified. As a result, 55 markets were identified in Chattogram, and 123 in Dhaka.

All markets identified in Chattogram, and some of those identified in Dhaka were recruited. In Dhaka, markets were classified as small (<10 vendors), medium (10-30 vendors) and large (>30 vendors). All large markets were recruited (n=10). In each administrative division in which markets were identified (26 out of 41 thanas), we randomly selected 30% of small markets (n=39) and 50% of medium markets (n=15). At least one small and/or medium market was selected in each thana where markets of each type were present. This was based on the assumptions that: (i) the smaller the market, the less variable the practices of vendors operating there, i.e. vendors' practices were more similar (1, 2) (ii) vendors operating in small markets were more likely to purchase poultry from the same set of larger markets (based on preliminary market visits and interviews).

All vendors operating in small and medium markets were recruited. This was not possible in large markets, given the high number of vendors operating there. In large markets, we randomly recruited ranged from 30 to 51 depending on the number of vendors. The sample size was computed in order to be 95% confident of identifying 90% of all possible origins of poultry sold at the market. The algorithm, detailed in Fournie et al. (1), was used with the following assumptions: (i) each vendor was assumed to purchase poultry from three different origins, (ii) each origin supplied an average of 5% of the vendors operating in the market (table S1).

Less than 1% of vendors refused to be interviewed, due to lack of time. Forty-three percent (Chattogram) to 65% (Dhaka) of mobile traders whose contact details were provided by interviewed vendors could not be enrolled, due to lack of time, or because they did not feel confident to answer questions by phone. The refusal rate was about 10% for face-to-face interviews with mobile traders recruited in the 6 markets in Dhaka.

1.1.2. Selection of feed dealers.

When interviewed, mobile traders purchasing poultry through feed dealers reported the upazila in which these feed dealers were based, but they were not asked about their contact details. Mobile traders who reported purchasing poultry from feed dealers based in upazilas near Dhaka and Chattogram were later contacted by phone and asked to provide the contact details of those feed dealers. As a result, 26 and 15 feed dealers were invited to take part in group discussions in Chattogram and Dhaka, respectively. Individual structured questionnaires were administered. Of these 41 feed dealers, 92.7% (n=38) were involved in the trade of broilers, 51.2% (n=21) in the trade of sonalis and 2.4% (n=1) in the trade of deshis. In order to recruit more feed dealers involved in the trade of sonalis and deshis, all interviewed mobile traders who had bought those types of chicken through feed dealers were contacted, and asked to provide contact details of feed dealers' who facilitated their poultry purchases. Overall, 80 feed dealers were interviewed, 72.5% (n=58) were involved in the trade of broilers, 51.3% (n=41) in the trade of sonalis, 7.5% (n=6) in the trade of deshis.

1.1.3. Additional investigations of deshis and ducks' trading patterns.

Mobile traders interviewed through the cross-sectional study who traded ducks and deshis hardly ever named farmers or feed dealers as their suppliers, but other mobile traders. However, they could not provide their contact details, just the upazila in which they operated. This meant that the sequence of actors involved in the trade of ducks and deshis was not captured. In order to address this gap, an additional cross-sectional survey was conducted in Chattogram from November 2017 to March 2018. Due to time constraints, the two Chattogram markets found to trade the largest numbers of ducks and deshis, during the main cross-sectional survey were selected. All vendors selling deshis and ducks (n=21) were asked about the contact details of

their suppliers. Forty-five mobile traders were thus identified. Four of them were interviewed to pilot the questionnaire, and 20 were purposively selected: (i) the 11 mobile traders trading ducks, 9 of whom also sold deshis, (ii) 5 mobile traders named by vendors operating in both markets – assuming that mobile traders supplying both surveyed markets were more likely to trade larger quantities and be supplied from more diverse origins, and (iii) 4 additional mobile traders randomly selected among the remaining ones. Two out of the 20 selected mobile traders refused to participate and were not replaced. The 18 interviewed mobile traders were asked about the numbers, origins and destinations of deshis and/or ducks they traded over the last two days they were operating. If poultry were bought from other vendors or mobile traders, their contact details were collected.

Through this 1st round of interviews, 41 mobile traders supplying the interviewed mobile traders were identified. Twenty-six traded deshis and 15 traded ducks. Half of the mobile traders trading ducks (n=8) and half of those trading deshis (n=13) were selected as follows:

- Mobile traders were grouped according to the district in which they operated and the type of poultry (deshi or duck) they traded.
- One deshi mobile traders was randomly selected for each district. This yielded a list of 11 deshi mobile traders. Two other deshi-trading mobile traders were randomly selected from the remaining ones to meet the objective of 13 participants.
- The same process was used to select 8 duck trading mobile traders.

Three mobile traders refused to participate, they were replaced by randomly selecting 3 other mobile traders, with respect to poultry type. All mobile traders interviewed during this 2nd round of interviews reported purchasing deshis and ducks from households or weekly rural markets. According to interviewed mobile traders and 10 deshi vendors operating in rural markets, 80% of people selling deshis and/or ducks in such markets were farmers, and 20% were mobile traders. Farmers sold their own and their neighbours' chickens. Mobile traders sold poultry they collected by bicycle from households within a 20-40km radius around those markets. It would usually take these mobile traders 1 to 2 days to collect enough poultry to sell at a rural market. We therefore assumed that deshis and ducks sold in rural markets originated from farms in the same union or upazila.

1.1.4. Longitudinal study.

This study focused on the trading patterns for the three main types of poultry sold in both cities: broiler, sonali and deshi chickens. The number of participants was defined according to available human and financial resources. Participants were selected among the vendors and mobile traders interviewed during the main cross-sectional study. We aimed to recruit the same numbers of vendors, mobile traders and feed dealers of each type in Dhaka and Chattogram but due to logistical constraints there were small variations in sample size between both cities. Feed dealers (n=19) who had facilitated the sales of the largest numbers of poultry were selected, ensuring that the main chicken types (i.e. broilers, sonalis as deshi trade was very rare for feed dealers) were equally represented. None of the feed dealers were lost to follow-up. Mobile traders (n=21 in Dhaka and n=16 in Chattogram) were randomly selected among those recruited during the main cross-sectional study and with respect to the type of traded chicken: 50% traded deshis, 25% traded broilers, and 25% traded sonalis. Four deshi mobile traders dropped out after the first month and were immediately replaced. Vendors were randomly selected from three purposively selected markets in each city (n=23 in Dhaka and n=20 in Chattogram). Markets were selected based on their size and the proportion of retailers operating there. None of the vendors were lost to follow-up. Fig. S1 presents a flowchart of the different surveys described here above.

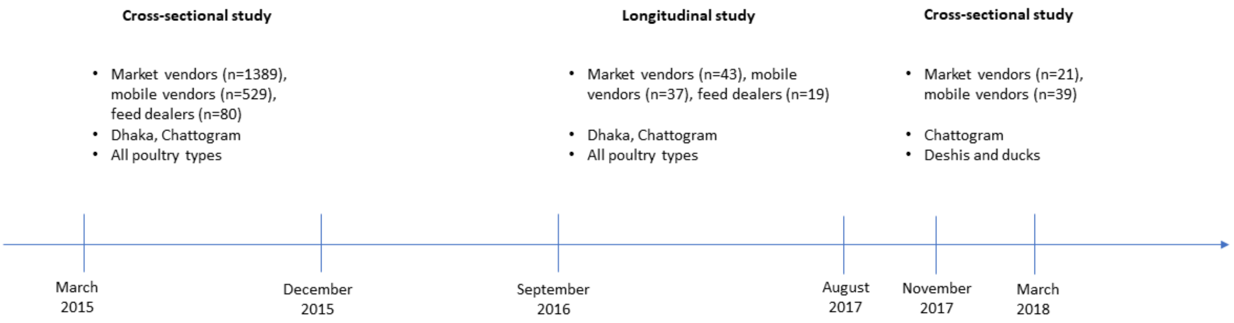

Fig. S1: Summary of studies carried out to collect data presented in this paper: type of study, type and number of participants, study area, poultry types considered, and time period for completion.

1.2. Data analysis

1.2.1. Longitudinal study.

Participants were interviewed every month over a year. We refer to an interview-period as the 4-day period for which each participant was asked about their trading practices in a given month. We aimed to assess whether the sales of broilers, sonalis and deshis by market vendors varied throughout the year. The analysis was conducted as a two-step process. First, we used logistic regression models with the sales of at least one chicken of a given type being sold during an interview-period as a binary outcome variable. Secondly, we used linear regression models with the daily number of chickens of a given type sold during an interview-period as the continuous outcome variable. For the latter, the number of chickens sold was log-transformed, and we only considered interview-periods for which at least one chicken, of a given type, was sold. We adjusted both models for each chicken type separately. As our objective was to explore the impact of the month on sales, we considered participants as fixed effects in the logistic and linear regression models, allowing us to control for unobserved individual characteristics (3).

1.2.2. Reconstruction of the network of transactions.

The algorithm presented below was applied to simulate the network of actors supplying a given poultry type *k* to a given study site, which could be either Dhaka or Chattogram.

i. Categories of actors, origins and destinations.

Vendors were differentiated into wholesalers and retailers, and mobile traders into different categories, according to their purchasing and selling practices for a given type of traded poultry, as explained in table S1 Vendors and mobile traders were classified such that the resulting network was acyclic, i.e. there was no transaction among actors of the same category (e.g. a vendor W1 did not buy/sell poultry to another vendor W1), and transactions were non-reciprocal between any two categories (e.g. a vendor W1 bought poultry from, but did not sell to mobile traders M1). Therefore, a bird could not be traded multiple time by the same actors (Table S2).

Each interviewed vendor sold poultry in one market, where they were interviewed, to *traders* or *end-users*. Information was unavailable about the category of these purchasing *traders* (i.e. mobile traders, vendors or non-market traders) and where these *traders* sold poultry. Interviewed mobile traders reported selling poultry to *vendors* or other *mobile traders* in markets. Vendors and mobile traders reported purchasing poultry from either *farmers*, *feed dealers*, *mobile traders* or *vendors*. The type of site and the geographical location (i.e. district or sub-district) of the transaction were reported. Farmers could either sell their poultry at their

farm gate, or at a market. There were rare instances where a vendor or mobile trader sold their own poultry flock. Therefore, an origin o or a destination d referred to a transaction involving (i) a given type of actor operating in (ii) a given type of site (e.g. farm, market) in (iii) a given geographical location.

Table S1: Categories of vendors and mobile traders. The number of vendors and mobile traders under each category is specified for each type of chickens.

Categories of vendors		Broiler	Sonali	Deshi
Retailer (R)	Sold all their poultry to end-users.	796 (91%)	457 (70%)	465 (79%)
1st-order wholesaler (W1)	Sold poultry to other traders. None of the purchased poultry transited through another market vendor's stall.	44 (5%)	21 (3%)	38 (6%)
2nd-order wholesaler (W2)	Sold poultry to other traders. Some poultry were bought directly or via a 1st order mobile trader, and none from 2nd and 3rd-order wholesalers.	20 (2%)	138 (21%)	56 (10%)
3rd-order wholesaler (W3)	Sold poultry to other traders. Some poultry were bought directly or via a mobile trader from a 2nd-order wholesaler.	13 (1%)	33 (5%)	30 (5%)
Categories of mobile traders				
1st-order mobile trader (M1)	Purchased all poultry from farmers, directly or via feed dealers.	174 (93%)	163 (84%)	35 (24%)
2nd-order mobile trader (M2)	Purchased poultry from 1st-order mobile traders, none from vendors.	6 (3%)	16 (8%)	99 (69%)
3rd-order mobile trader (M3)	Purchased at least some poultry from vendors.	8 (4%)	16 (8%)	10 (7%)

Table S2: Possible transactions between the different categories of actors. A grey matrix cell indicates that poultry can be sold from a given category of actors (in rows) to another category of actors (in columns). For instance, mobile traders M1 can sell poultry to retailers, but retailers cannot sell poultry to mobile traders M1. non-M Tr.: non-market traders (traders purchasing poultry from market vendors and selling these outside markets)

FROM \ TO	Farm	Feed dealer	M1	M2	M3	W1	W2	W3	Retailer	End-users	non-M Tr.
Farm		■	■	■	■	■	■	■	■		
Feed dealer			■	■	■	■	■	■	■		
M1				■	■	■	■	■	■		
M2					■	■	■	■	■		
M3							■	■	■		
W1					■		■	■	■	■	■
W2								■	■	■	■
W3									■	■	■
Retailer										■	
End-users											
non-M Tr.											

ii. Number of poultry bought and sold by interviewed groups of vendors and mobile traders.

A simulation started by defining, for each actor i, the number s_{ik}^{*} of poultry of type k they sold in a week, drawn from a uniform distribution with lower and upper bounds the range of values they reported selling. Similarly, the number b_{iko} (s_{ikd}) of poultry they bought (sold) from each origin o (destination d) was drawn from a uniform distribution with lower and upper bounds the minimum and maximum number of poultry they reported having bought (sold) from that

origin. As b_{iko} and s_{ikd} were generated through independent simulations, they were then adjusted as $b_{iko}s^*_{ik}/\sum_o b_{iko}$ and $s_{ikd}s^*_{ik}/\sum_d s_{ikd}$.

B_{lcko} (S_{lckd}) was the number of poultry of type k purchased from (sold to) an origin o (a destination *d*) by all vendors of category *c*, with $c \in \{R, W_1, W_2, W_3\}$, operating in a market *l*:

$$B_{lcko} = \sum_{i,i\in V_{lc}} b_{iko} \text{ and } S_{lckd} = \sum_{i,i\in V_{lc}} s_{ikd}$$

Where V_{lc} referred to the subset of vendors of category c operating in a market *l*. While all vendors operating in Chattogram and small and medium Dhaka markets were recruited, a random sample of vendors were interviewed in large Dhaka markets. In such markets, the sample size was accounted for by dividing B_{lcko} and S_{lckd} by the proportion of interviewed vendors.

iii. Transactions between interviewed actors.

We refer to a network link $T^{lk}_{i\to j}$ as the number of poultry of type *k* traded in a transaction location *l* from a node *i* to a node *j*. A transaction location was either (1) a given market or (2) farms in an upazila. L was the set of all possible locations, i.e. $l \in L$. A node was either (1) all end-users purchasing poultry from vendors in a given market, (2) all non-market traders purchasing poultry from vendors in a given market and selling these outside markets, (3) an individual mobile trader, (4) all vendors of category *c* (i.e. R, W1, W2, W3) operating in a given market, (5) feed dealers in a an upazila, (6) farms in an upazila. We sequentially reconstructed network links between the available set of nodes, from the sinks (i.e. end-users) up to the origins (i.e. farms).

1) Transactions from vendors to end-users in a market *l*. It was expressed as $T^{l,k}_{i=v_{lc}\to j=end-users_l} = S_{l,c,k,d=end-users_l}$. $i = v_{lc}$ referred to node *i* being the group of vendors of category *c* operating in a location *l*.

2) Purchases in location *l* by retailers operating in a market *y*. The proportion of poultry purchased by retailers operating in market *y* from a given origin o was $\alpha^o_{y,c=R,k} = B_{y,c=R,k,o}/\sum_{q,q\in O} B_{y,c=R,k,q}$, where *O* was the set of possible origins. As mentioned above, an origin *o* could be either (1) farms in an upazila, (2) feed dealers in an upazila, (3) mobile traders, (4) wholesalers:

2.1) Farms: $T^{l,k}_{i=l\to j=v_{y,c=R}} = \alpha^{o=l}_{y,c=R,k} \sum_j T^{y,k}_{i=v_{y,c=R}\to j}$, with *l* being the location of the farms. If only the district was known, the number of poultry reported to have been purchased was equally distributed among all upazilas in the district.

2.2) Feed dealers: $T^{l,k}_{i=l\to j=v_{y,c=R}} = \alpha^{o=l}_{y,c=R,k} \sum_j T^{y,k}_{i=v_{y,c=R}\to j}$, with *l* being the location of the feed dealers' stores.

2.3) Mobile traders: The proportion of poultry bought by vendors (here retailers) from an interviewed mobile trader *i* in location *l* was the ratio between the number of poultry sold by this mobile trader, and all interviewed mobile traders, to vendors in this location: $\gamma^v_{i,k,l} = s_{i,k,d=v_l}/\sum_{i,i\in M_l} s_{i,k,d=v_l}$. $d = v_l$ meant that poultry were sold to vendors in location *l*, and M_l was the subset of interviewed mobile traders supplying vendors in *l*. The number of poultry traded between an interviewed mobile trader *i* and retailers in market *y* was: $T^{l,k}_{i\to j=v_{y,c=R}} =$

$\gamma_{i,k,l}^{v}\alpha_{y,c=R,k}^{o=m_l}\sum_{j}T_{i=v_{y,c=R}\to j}^{y,k}$. $o=m_l$ meant that retailers bought poultry from mobile traders in l.

2.4) Wholesalers: The proportion of poultry bought by vendors (here retailers) from a group of wholesalers, characterised by their category c_i and their market l_i (with $l_i = l$ as wholesalers only sold in their market) was the ratio between the number of poultry sold by that category, and all categories of wholesalers, in l: $\omega_{i,k}^{v} = S_{l,c=c_i,k,d=T_l}/\sum_{x,x\in\{W_l,W_2,W_3\}} S_{l,x,k,d=T_l}$. $d = T_l$ meant that poultry were sold to traders in l, as wholesalers did not distinguish the categories of traders they sold to. The number of poultry traded from wholesalers of category c_i to retailers was then: $T_{i\to j=v_{y,c=R}}^{l,k} = \omega_{i,k}^{v}\alpha_{y,c=R,k}^{o=W_l}\sum_{j}T_{i=v_{y,c=R}\to j}^{y,k}$. $o = W_l$ meant that retailers bought poultry from wholesalers in l.

3) Purchases in location l by wholesalers of category c operating in market y. It was estimated in the same way as for retailers, except that (i) wholesalers W_1 were only supplied by mobile traders classified as M_1 or M_2, and (ii) no wholesalers purchased poultry from other wholesalers of the same category, nor from W_3.

4) Transactions in a market l from wholesalers to non-market traders. The number of poultry sold to non-market traders was the difference between (i) the number of poultry that wholesalers of category c operating in location l had reported selling to *traders* ($S_{l,c,k,d=T_l}$) and (ii) the number of poultry that was purchased, from that group of wholesalers, by other vendors and mobile traders:

$$\forall c \in \{W_1, W_2, W_3\},\quad T_{i=v_{l,c}\to j=non-M\,Tr_l}^{l,k} = S_{l,c,k,d=T_l} - \sum_{x,x\in\{W_2W_3,R\}} T_{i=v_{l,c}\to j=v_{l,x}}^{l,k} - \sum_{j,j\in M_l} T_{i=v_{l,c}\to j}^{l,k}$$

5) Purchases in location l by a mobile trader. The proportion of poultry a mobile trader m purchased from an origin o was expressed as $\theta_{m,k}^{o} = b_{m,k,o}/\sum_{q,q\in O} b_{m,k,q}$. The origin o could be (1) farms in an upazila, (2) feed dealers in an upazila, (3) other mobile traders, or (4) wholesalers.

5.1) Farms: $T_{i=l\to j=m}^{l,k} = \theta_{m,k}^{o=l}\sum_{z,j}T_{i=m\to j}^{z,k}$

5.2) Feed dealers: $T_{i=l\to j=m}^{l,k} = \theta_{m,k}^{o=l}\sum_{z,j}T_{i=m\to j}^{z,k}$

5.3) Mobile traders: The proportion of poultry bought by a mobile trader m in location l from a given mobile trader i was equal the ratio between the number of poultry sold in location l by mobile trader i and all other interviewed mobile traders, $\gamma_{i,k,l}^{mm} = s_{i,k,d=mm_l}/\sum_{i,i\in M_l} s_{i,k,d=mm_l}$. $d = mm_l$ meant that poultry were sold to mobile traders in a location l, and M_l referred to the subset of interviewed mobile traders in location l. As with wholesalers, mobile traders included in M_l was constrained by their category (Tables S1 and

S2). The number of poultry traded from an interviewed mobile trader *i* to a mobile trader *m* was then: $T^{l,k}_{i \to j=m} = \gamma^{mm}_{i,k,l} \theta^{o=m_l}_{m,k} \sum_{z,j} T^{z,k}_{i=m \to j}$

5.4) Wholesalers: $T^{l,k}_{i \to j=m} = \theta^{o=W_l}_{m,k} \sum_{z,j} T^{z,k}_{i=m \to j}$. $o = W_l$ meant that poultry were sold by wholesalers in a location *l*.

6) Transactions involving feed dealers. Feed dealers facilitated poultry sales from farmers to mobile traders and vendors. As explained in the main text, we estimated, out of all poultry farms for which feed dealers facilitated transactions, the proportion of farms that was located (i) in the same upazila as the feed dealer's store, P_1, (ii) in the same district but a different upazila, P_2, (iii) in a neighbouring district, P_3. Let's δ represent feed dealers in a given upazila *w*. The number of poultry traded from farms *i* in any upazila *u* was defined as follows:

6.1) $u = w$, $T^{l=i,k}_{i \to \delta} = P_1 \sum_j T^{l=\delta,k}_{\delta \to j}$. $\sum_j T^{l=\delta,k}_{\delta \to j}$ referred to the total number of poultry sold by feed dealers δ to all other possible nodes.

6.2) $u \neq w$ but *u* was in the same district as *w*, $T^{l=i,k}_{i \to \delta} = P_2 \sum_j T^{l=\delta,k}_{\delta \to j} / (n_u - 1)$, where n_u was the number of upazilas in the district of *u*.

6.3) *u* was in a district neighbouring the district of *w*, $T^{l=i,k}_{i \to \delta} = P_3 \sum_j T^{l=\delta,k}_{\delta \to j} / n_d$, where n_d was to the number of upazilas in all the districts neighbouring the district of *u*.

If only the district of the feed dealer's store was reported, for any upazila *u* in that district $T^{l=i,k}_{i \to \delta} = (P_2 + P_1) \sum_j T^{l=\delta,k}_{\delta \to j} / n_u$.

A very small number of vendors and mobile traders reported trading poultry from their own farm. The locations of those farms were, however, not captured. We assumed that vendors' farms were located in the upazila of the market where they operated, and mobile traders' farms were located in the upazila where they had purchased the largest proportion of their poultry from (hypothesising that mobile traders preferentially purchased poultry closest to their home, as observed with feed dealers).

iv. Transaction involving non-surveyed markets in Dhaka.

Trading practices of vendors operating in non-surveyed Dhaka markets were imputed based on the practices of vendors operating in surveyed markets. Each non-surveyed market was associated to a surveyed market, referred to as a *reference* market. It was randomly selected among the surveyed markets of the size category [i.e. small (<10 vendors), medium (10-30 vendors), large (>30 vendors)] and located in the same thana (i.e. first administrative division under city corporations) as the non-surveyed market. If none of the surveyed markets matched those criteria, a reference market was randomly selected among the surveyed markets of the size category in the same city corporation (Dhaka North or South) as the non-surveyed market. In a non-surveyed market *z*, the number of poultry sold to end-users and non-market traders, and the number and origins of poultry purchased by different categories of vendors were imputed based on trading practices reported in the selected *reference* market *r*.

First, we created as many categories of vendors (R, W_1, W_2, W_3) in z as there were in r. Then, we estimated the number of poultry sold to customers, and purchased from each origin, by each category of vendors, considering the following scenarios.

1) None of the interviewed mobile traders reported selling poultry to vendors in market z. The numbers of poultry sold to end-users and non-market traders in market z by each category c of vendors were the same as in LBM r, e.g. $T^{z,k}_{i=v_{z,c} \to j=end-users_z} = T^{r,k}_{i=v_{r,c} \to j=end-users_r}$.
If vendors operating in the reference market r purchased poultry from other vendors in this market, in other terms, if some transactions only involved vendors from r, those transactions were repeated, involving the same categories of vendors and the same number of poultry, but in market z: $T^{z,k}_{i=v_{z,t_1} \to j=v_{z,t_2}} = T^{r,k}_{i=v_{r,t_1} \to j=v_{r,t_2}}$, where t_1, t_2 represent two different categories of vendors (e.g. $t_1 = W_1$ and $t_2 = R$).
If vendors operating in market r bought poultry from a mobile trader i, another mobile trader was created, such that they sold poultry to vendors in z from the same suppliers as i. Practically, it was the same as considering that mobile trader i, also supplied vendors in z, i.e. $T^{z,k}_{i \to j=v_{z,c}} = T^{r,k}_{i \to j=v_{r,c}}$.
If vendors in market r purchased poultry in another location ($l \neq r$), the origins and number of poultry purchased by each category c of vendors in market z were the same as for vendors of the same category in LBM r, i.e. $\forall c \in \{R, W_1, W_2, W_3\}, \quad T^{l,k}_{i \to j=v_{z,c}} = T^{l,k}_{i \to j=v_{r,c}}$.

2) Some interviewed mobile traders reported selling poultry to vendors in market z. A total of 13 interviewed mobile traders reported selling poultry to stallholders in 6 non-surveyed markets: 3 sold broilers in 2 non-surveyed markets, 7 sold sonalis in 4 markets, and 3 sold deshis in 2 markets. In such cases, all vendors in market z were assumed to be retailers (as in most small surveyed markets), and other vendor categories were ignored. Thus, situations where, for example, a wholesaler classified as W_1 (imputed based on the composition of the vendor population in the reference market) purchased from a mobile trader classified as M_3 were avoided.
The number of poultry sold by retailers operating in LBM z to end-users was equal to the number of poultry sold to customers by all stallholders operating in LBM r:

$$T^{z,k}_{i=v_{z,c=R} \to j=end-users_z} = \sum_{c \in \{R, W_1, W_2, W_3\}} T^{r,k}_{i=v_{r,c} \to j=end-users_r} + \sum_{c \in \{W_1, W_2, W_3\}} T^{r,k}_{i=sh_{r,c} \to j=non-M\,Tr_r}$$

The number of poultry purchased by retailers operating in market z from each mobile trader m who reported selling poultry to vendors in market z: $T^{z,k}_{m \to j=v_{z,c=R}} = \gamma^{v}_{m,k,z} T^{z,k}_{i=v_{z,c=R} \to j=end-users_z}$
Where $\gamma^{v}_{m,k,z}$ was the proportion of poultry bought by vendors (here retailers) from a given mobile trader m in market z. If only one mobile trader reported selling poultry to vendors in market z, then $\gamma^{v}_{m,k,z} = 1$.

v. Transactions for which sales, but no purchases, were reported.

In a small number of instances (3, 2, and 3 mobile traders selling broilers, sonalis and deshis in Dhaka, and 13 and 6 mobile traders selling sonalis and deshis in Chattogram), a mobile trader m reported selling poultry of type k to vendors in a surveyed market z, but none of the interviewed vendors reported purchasing that type of poultry from mobile traders. This might be due to infrequent sales/purchases, as both mobile traders and vendors were asked about their transaction in the week preceding the interview, but the interview periods did not overlap.

If some vendors in market *z* traded poultry of type *k* (but did not mention buying those from a mobile trader), a proportion ζ of vendors' purchases was attributed to mobile trader m: $\forall c \in H,\ \ T^{z,k}_{m\to j=v_{z,c}} = \zeta \sum_j T^{z,k}_{i=v_{z,c}\to j}$, with H being the categories of vendors to be considered, $H = \{R, W_1, W_2, W_3\}$ or $H = \{R, W_2, W_3\}$ if m was classified as M_3. In order for the number of poultry traded in market *z* to remain unchanged, purchases from any other origin *i* were adjusted to $(1-\zeta)T^{z,k}_{i\to j=v_{z,c}}$. ζ was set to 0.05, hypothesising that if vendors did not mention this origin of poultry, it was infrequent and represented a small proportion of their purchases.
If none of the interviewed vendors in market *z* reported trading poultry of type *k*, transactions between a mobile trader *m* and retailers in *z* were created. A new node of retailers in market *z* was thus created. We identified a *reference* market *r*, as described above, and retailers from *z* were assumed to sell as many poultry of type *k* to end-users as retailers did in *r*: $T^{z,k}_{i=v_{z,c=R}\to j=end-users_z} = T^{r,k}_{i=v_{r,c=R}\to j=end-users_r}$. Sales from a mobile trader *m* to retailers in *z* were: $T^{z,k}_{m\to j=v_{z,c=R}} = \gamma^{v}_{m,k,z} T^{z,k}_{i=v_{z,c=R}\to j=end-users_z}$, where $\gamma^{v}_{m,k,z}$ was the proportion of poultry sold to vendors in *z* by a given mobile trader *m*.

vi. Transactions in Dhaka and Chattogram for which purchases, but no sales, were reported.

Some suppliers reported by interviewees did not correspond to any interviewed actor. For instance, a vendor may have reported purchasing chickens from mobile traders in market *l*, but no mobile trader operating in that market was interviewed. We imputed the practices of those non-interviewed suppliers by considering the following scenarios.

1) Wholesalers supplying vendors. Interviewed vendors of category *c* in market *y* ($v_{y,c}$) reported purchasing poultry from wholesalers in a market *l*, but none of the interviewed wholesalers reported selling poultry to *traders* in that market. We created a node $i = v_{l,x}$ corresponding to wholesalers of unknown category *x* operating in market *l* selling poultry to $v_{y,c}$:
$\forall c \in \{R, W_2, W_3\},\ \ T^{l,k}_{i=v_{l,x}\to j=v_{y,c}} = \alpha^{o=W_l}_{y,c,k} \sum_j T^{y,k}_{i=v_{y,c}\to j}$, where $\alpha^{o=W_l}_{y,c,k}$ was the proportion of poultry traded by vendors $v_{y,c}$ which were supplied by wholesalers operating in market *l*.
The category *x* of $v_{l,x}$ and the origins of their poultry were imputed by identifying a *reference* surveyed market *r*. In addition to the criteria mentioned in section 1.2.1 iv. for the selection of *reference* markets, only markets with categories of wholesalers compatible with $v_{y,c}$ were considered. For example, if $v_{y,c}$ was classified as $c = W_2$, the set of possible *reference* LBMs was restricted to those where W_1s operated.
Once a *reference* market *r* selected, as many categories of wholesalers in market *l* were created as there were in markets *r*, and transactions between each of these nodes and $v_{y,c}$ were:
$\forall v \in H\ \ T^{l,k}_{i=v_{l,v}\to j=v_{y,c}} = \left(\sum_d S_{r,v,k,d} / \sum_{d,q,q\in H} S_{r,q,k,d}\right) T^{l,k}_{i=v_{l,x}\to j=v_{y,c}}$, with H being the categories of wholesalers to be considered. For instance, if $c = W_2$, $H = W_1$. Origins of poultry purchased by newly created wholesalers where imputed as follows.

- If wholesalers in the *reference* market *r* purchased poultry from other wholesalers in that market, similar transactions were reproduced in market *l*.

- If wholesalers in *r* purchased poultry from wholesalers in another location *z*, wholesalers in *l* were then assumed to purchase poultry from the same suppliers in that location *z*.

- If wholesalers in *r* bought poultry from a mobile trader *m*, another mobile trader buying poultry from the same suppliers as *m*, and supplying wholesalers in *l*, was created. Practically, it was the same as considering that *m*, who supplied wholesalers in *r*, also supplied wholesalers in *l*.

2) Wholesalers supplying mobile traders. By definition, these wholesalers were necessarily W_1 (Table S2). We created a node $i = v_{l,x=W_1}$ corresponding to wholesalers selling poultry to an interviewed mobile trader *p*, with transactions: $T^{l,k}_{i=v_{l,x=W_1}\to j=p} = \theta^{o=v_{l,x=W_1}}_{p,k} \sum_{j,y} T^{y,k}_{i=p\to j}$, where $\theta^{o}_{p,k}$ was the proportion of poultry traded by *p* which were supplied by a given origin *o*. The procedure to identify the suppliers of $v_{l,x=W_1}$ was similar to the one described above.

3) Mobile traders supplying vendors. Interviewed vendors of category *c* in market *y* ($v_{y,c}$) reported purchasing poultry from mobile traders in a market *l*, but none of the interviewed mobile traders reported selling poultry in that market. A *reference* market *r* was identified to impute the practices of the supplying mobile trader(s). The possible set of *reference* markets was restricted to markets with categories of mobile traders compatible with $v_{y,c}$. Once the *reference* market *r* identified, as many mobile traders were created in market *l* as there were in market *r*. The suppliers of those newly created mobile traders were the same as mobile traders in *reference* market *r*. Practically, it was the same as considering that mobile traders supplying vendors in *r* also supplied vendors in *l*.
$\forall m \in M_r \quad T^{l,k}_{i=m\to j=v_{y,c}} = \left(\sum_j T^{r,k}_{i=m\to j} / \sum_{j,q,q\in M_r} T^{r,k}_{i=q\to j}\right) \alpha^{o=m_l}_{y,c,k} \sum_j T^{y,k}_{i=v_{y,c}\to j}$, with M_r the set of mobile traders in *r*.

4) Mobile traders supplying mobile traders. A *reference* market *r* was identified as described above, and for each mobile trader *m* selling poultry in this market: $\forall m \in M_r \quad T^{l,k}_{i=m\to j=p} = \left(\sum_j T^{r,k}_{i=m\to j} / \sum_{j,q,q\in M_r} T^{r,k}_{i=q\to j}\right) \theta^{o=m_l}_{p,k} \sum_{j,y} T^{y,k}_{i=p\to j}$, with M_r was the set of mobile traders in *r* and $\theta^{o}_{p,k}$ the proportion of poultry a mobile trader *p* purchased from a given origin *o*.

vii. Transactions outside Dhaka and Chattogram.

Some interviewees purchased chickens from wholesalers and/or mobile traders outside Dhaka and Chattogram. We imputed the practices of those non-interviewed suppliers by considering the following scenarios.

1) Wholesalers supplying vendors. Vendors of category *c* in market *y* ($v_{y,c}$) were supplied by a wholesaler operating in market *l*, outside the study area. Outside of Dhaka and Chittagong cities, markets were generally located in rural or semi-urban areas. The cross-sectional study was also conducted in such a sub-district (Raozan, in Chattogram district). A total of 128 market vendors were interviewed, and wholesalers classified as W_2 were rare (<2% of broilers' movements) or absent (none were involved in the trade of sonalis and deshis). We therefore assumed that wholesalers outside of the study area were of category W_1. We created a node $i = v_{l,x=W_1}$ corresponding to wholesalers in market *l* selling poultry to vendors $v_{y,c}$: $T^{l,k}_{i=v_{l,x=W_1}\to j=v_{y,c}} = \alpha^{o=W_l}_{y,c,k} \sum_j T^{y,k}_{i=v_{y,c}\to j}$, where $\alpha^{o}_{y,c,k}$ was the proportion of poultry traded by vendors $v_{y,c}$ which was supplied by a given origin *o*. As hardly any of the interviewed wholesalers purchased poultry directly from farms, we hypothesised that wholesalers of category W_1 operating in markets located outside of the study area were supplied from farms

via mobile traders. We therefore created a node $i = m$, corresponding to a mobile trader *m* of category M_1 who supplied poultry to wholesalers $v_{l,x=W1}$: $T^{l,k}_{i=m \to j=v_{l,x=W1}} = T^{l,k}_{i=v_{l,x=W1} \to j=v_{y,c}}$. The number of poultry purchased by mobile trader *m* from farms in different upazilas was simulated as detailed in section 1.2.1 iii., with the proportion of chickens sourced from the same district as market *l* and neighbouring districts were $(P_1 + P_2)$ and P_3, respectively.

2) Wholesalers supplying mobile traders. A mobile trader *p* was supplied by wholesalers in market *l*, outside the study area. We created a node $i = v_{l,x=W_1}$ corresponding to those wholesalers, the transaction was: $T^{l,k}_{i=v_{l,x=W1} \to j=p} = \theta^{o=v_{l,W_1}}_{p,k} \sum_{j,y} T^{y,k}_{i=p \to j}$, with $\theta^{o=v_{l,W_1}}_{p,k}$ the proportion of poultry traded by mobile trader *p* which was supplied by a given origin *o*. Likewise, a mobile trader supplying wholesalers in *l*, and purchasing chickens from farms in the district of *l* and neighbouring districts was created as described above.

3) Mobile traders supplying vendors. We created a node $i = m$ corresponding to mobile traders selling poultry to vendors $v_{y,c}$ in market *l*. Transactions were: $T^{l,k}_{i=m \to j=v_{y,c}} = \alpha^{o=m_l}_{y,c,k} \sum_{j} T^{y,k}_{i=v_{y,c} \to j}$, with $\alpha^{o}_{y,c,k}$ the proportion of poultry traded by vendors $v_{y,c}$ which was supplied by a given origin *o* The number of poultry that mobile trader *m* purchased from farms in different upazilas was then estimated as described in paragraph 1) of this section.

4) Mobile traders supplying mobile traders. A node $i = m$ was created, corresponding to mobile traders selling poultry to a mobile trader *p* in market *l*. Transactions were: $T^{l,k}_{i=m \to j=p} = \theta^{o=m_l}_{p,k} \sum_{j,l} T^{l,k}_{i=p \to j}$, with $\theta^{o}_{p,k}$ the proportion of poultry traded by mobile trader *p* which was supplied by a given origin *o*. The number of poultry that mobile trader *m* purchased from farms in different upazilas was then estimated as described in paragraph 1) of this section.

5) Village markets supplying mobile traders. If a mobile trader *p* (no wholesalers did) purchased deshis from a local, rural market held weekly, they did not report whether the seller was a farmer or another mobile trader. Informal discussions with mobile traders and visits of such markets suggested that about 80% of deshis were sold by local farmers and 20% by local mobile traders who purchased chickens within a small radius (<30km) around the market. Therefore, if a mobile trader was supplied in a village market, the following transactions were created:
- a transaction from farms, in the same upazila as the village market, to mobile trader *p*. It accounted for 80% of the purchases made by mobile trader *p* in the village market.
- a transaction from a mobile trader *m* operating at the village market and mobile trader *p*, and a transaction from farms, in the same upazila as the village market, to mobile trader *m*. These transactions accounted for 20% of the purchases made by mobile trader *p* at the village market.

viii. Adjusting transactions.

The imputation of missing data resulted in some groups of vendors and mobile traders selling more poultry than they purchased. The number of poultry traded through each transaction T was adjusted to ensure that each actor, or group of actors, purchased as many poultry as they sold. It was conducted sequentially, first the retailers (i.e. R), then groups of traders classified as W_3, W_2, M_3, W_1, M_2 nd M_1. For a given sequence, and each node *h* – a group of vendors of a given category operating in a given market, or a given mobile trader – the ratio sales-purchases was defined as follows:

$$r_{h,k} = \sum_{y,j} T_{h \to j}^{y,k} \Big/ \sum_{y,i} T_{i \to h}^{y,k}$$

and transactions corresponding to the purchase of poultry by node h were adjusted:
$\forall i, l \;\; T_{i \to h}^{l,k*} = r_{h,k} T_{i \to h}^{l,k}$.

1.2.3. Duration that chickens spent with market vendors

We estimated the distribution of *marketing time*, which was defined as the time spent by chickens in vendors' flocks in a market. Marketing time was estimated in order to assess the potential for viral amplification to take place in markets. For each chicken type and city supplied, we simulated the trajectory of a chicken among actors using the algorithm detailed in section 1.2.3 (Sequences of actors and chicken population dynamics). We assessed the length of time that the chicken spent with vendors in Dhaka or Chattogram markets along the simulated sequence of actors. For any vendor, it was the sum of:

- The *selling period*, defined as the length of time during which a chicken was offered for sale.
- The *storing period*, defined as the length of time during which a chicken was stored before being offered for sale.

i. Selling period.

1) The daily number of chickens sold and bought by the vendor. To do so, sales and purchases of the vendor of interest were simulated over 10 weeks. For any week w, the number of chickens sold by the vendor was randomly sampled from an uniform distribution: $S_w \sim U[\min\_s, \max\_s]$, with $\min\_s$ and $\max\_s$ being the minimum and maximum number of chickens the vendor reporting selling in a week. The number s_d of chickens sold on day d in week w was simulated with a multinomial trial, with S_w as the number of trials and equal probabilities for chickens to be sold on any day of the week. The days on which chickens were purchased was defined by the frequency of purchases reported by vendors: everyday, every other day or every 3 days. The number b_d of chickens bought on a day d could then be estimated: if chickens were purchased every day: $b_d = s_d$, every over other day: $b_d = s_d + s_{d+1}$ every 3 days: $b_d = s_d + s_{d+1} + s_{d+2}$.

2) The *cohort* through which a chicken was sold. A *cohort* was defined as the group of chickens offered for sale between 2 successive purchases. For instance, if a given chicken was unsold by the time newly purchased chickens joined a vendor's flock and was reoffered for sale with this new group of chickens, it was considered to join a new cohort. For each day during which a vendor purchased chickens, the presence of chickens left unsold from the former cohort was simulated through a binomial trial with a probability of success ς, the surplus frequency reported by the interviewed vendor, defined as the ratio between the number of days a week a vendor reported having surplus and the number of days a week the vendor purchased chickens. For a chicken purchased on day d in week w, we simulated whether a given chicken was sold by the time the vendor purchased new chickens. If there was no simulated surplus, all chickens were sold by the next purchase. If there was some surplus, the number of chickens left unsold was simulated as: $\lambda_d \sim U[\min\_\lambda, \max\_\lambda]$, with $\min\_\lambda$ and $\max\_\lambda$ being the minimum and the maximum number of chickens the vendor reported having as surplus, when they had surplus. The presence of a given chicken among those left unsold was simulated through a binomial trial, with probability of success $\lambda_d/(\lambda_d + b_d)$.
If the given chicken was left unsold and the vendor reported prioritising the sale of chickens left unsold, then that chicken was sold as part of the following cohort. In that case, the number of cohorts the chicken transited through, as part of the vendor's flock, was $c = 2$.

If the vendor did not report prioritising the sale of chickens left unsold, any chicken had the same probability to be sold regardless of the time it had already spent in the vendor's flock; the occurrence of surplus, and the presence of a given chicken within this surplus, were repeatedly simulated, as described above, until identifying the number of cohorts c through which a given chicken transited through before being sold.

3) Time at which a chicken was sold. The selling period was $f(c-1)+\theta$. $(c-1)$ was the number of cohorts that a chicken went through without being sold. f was the duration, in hours, between 2 consecutive cohorts. It related to the supply frequency: if a vendor purchased chickens every day, $f=24$, every other day, $f=48$. θ was the difference between the time at which chicken was sold, and the time at which it was offered for sale as part of its last cohort. The time at which a chicken was sold was randomly selected within the period of time during which a cohort was offered for sale (as reported). The probability of a chicken being sold remained constant for the period during which it was offered for sale. The way in which this period was defined depended on the vendor's supply practices. We considered the following scenarios (Fig. S2):

- Scenario 1: Chickens were purchased when the stall was closed: a cohort was first offered for sale as the stall re-opened, and last offered for sale when the stall last closed before receiving a new batch of chickens.
- Scenario 2: Chickens were purchased during stall opening hours, ≤3 hours before stall closure: a cohort was first offered for sale on the following day it was bought (i.e. the next time the stall re-opened). It was last offered for sale when the stall closed on the day that a new cohort of birds was received.
- Scenario 3: chickens were purchased during stall opening hours, ≤3 hours after stall opening: a cohort was first offered for sale when chickens were received at the stall, or, for chickens left unsold, when the stall opened on that day. The cohort was last offered for sale when the stall closed on the day preceding the arrival of newly purchased chickens.
- Scenario 4: chickens were purchased during stall opening hours, >3 hours after stall opening and >3 hours before stall closure: a cohort was first offered for sale when chickens were received at the stall, and it was last offered for sale with the arrival of newly purchased chickens.

Note that the timing of stall closure and opening may depend for some vendors on the destination of the chicken for which the trajectory among network actors was simulated, i.e. an *end-user* or another *trader*. The time at which a given chicken was purchased by a vendor was defined by randomly selecting a time within the range of hours that the vendor reported buying at, from a type of supplier (i.e. another vendor, a mobile trader, a farm) compatible with the simulated sequence of actors through which chickens were traded. If, for a given type of supplier, chickens could enter the vendor's flock at different time slots, one time slot was randomly selected.

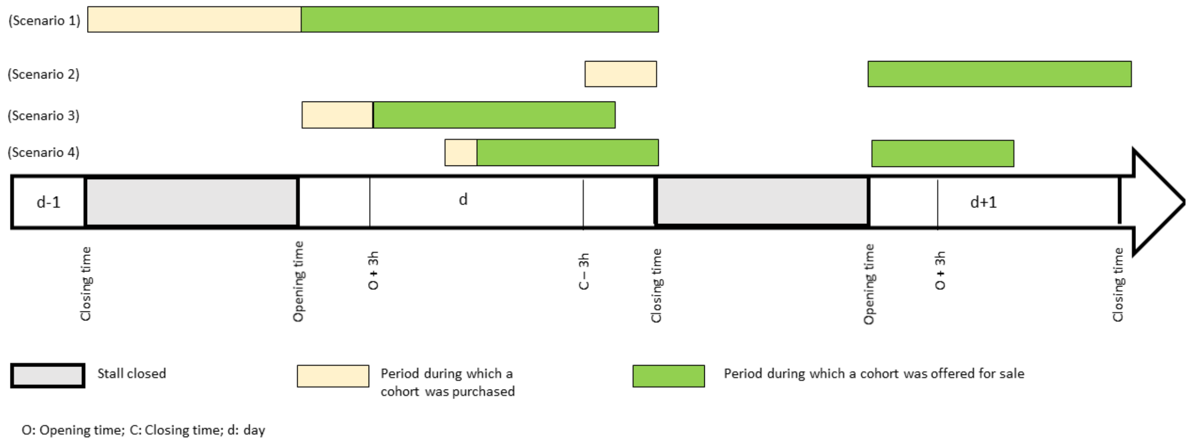

Fig. S2: Periods during which chickens were first and last offered for sale, according to when they were supplied. Here, chickens were assumed to be purchased and sold daily.

ii. The storing period.

Depending on the timing of the purchase, a chicken might not have been offered for sale as soon as it joined a vendor's flock (e.g. it was purchased when the stall was closed). It would then be stored for a given period, referred to as the storing period. The storing period was then the difference between the time at which a chicken was offered for sale for the first time by the vendor, and the time at which a chicken was actually purchased by this vendor.

iii. Marketing time.

The marketing time for a given chicken along the simulated sequence of actors was the sum of the selling period and storing period. The market-level marketing time was then defined according to the composition of the simulated sequence of actors:
- If the sequence of actors included only one vendor, or several vendors but all of them operated in different markets, there was as many simulated market-level marketing times as there were simulated vendor-level marketing times.
- If the sequence of actors included several vendors trading with one another in the same market, the marketing times simulated for each of those vendors were summed up to give the market-level marketing time.

This algorithm was repeated 100,000 for each chicken type and city supplied. The distribution of simulated marketing times provided an estimation of the probability of a chicken of a given type remaining in a market in Dhaka or Chattogram as a function of time.

1.2.4. AIV transmission dynamics.

We provide here additional information to complement the description of the within-market transmission model presented in the main text. The model is similar to (1), except that the lengths of the pre-infectious and infectious periods were the same for all chickens. In other words, all pre-infectious chickens spent the same number of hours T_E in the pre-infectious compartment before becoming infectious, and all infectious chickens spent the same number of hours T_I in the infectious compartment before recovering or dying of the infection.
The marketed chicken population was divided into four compartments: susceptible $S_{t,d}$, pre-infectious $E_{t,d}$, infectious $I_{t,d}$ and recovered (or removed) $R_{t,d}$. The index t referred to the number of hours since the last supply. d referred to the number of days that these chickens had been offered for sale at the market. As mentioned in the main text, the same number of chickens entered in the market each day, all at the same time. The proportions of infectious and pre-

infectious chickens among daily newly introduced chickens were P_{intr} and $P_{intr}T_E/T_I$, with P_{intr} being the prevalence of infectious chickens in the farmed population.
The number of chickens present in the market at time t, and remaining there at time $t+\Delta t$, was modelled as a binomial process with 1 – [probability of sales] as the probability of success, and the number of chickens in each compartment at time t as the number of trials. The probability of a chicken being sold between times t and $t+\Delta t$ depended on the length of time it had already spent in vendors' stalls at the market. The number of chickens becoming infected at time $t+\Delta t$ was also modelled as a binomial process, with the probability of infection $p_t = 1 - exp[-\tau(I_t + \eta C_t)\Delta t]$ as the probability of success, and the number of susceptible chickens that were not sold between times t and $t+\Delta t$ as the number of trials. The number of infectious chickens at time t, denoted I_t, was actually $\sum_d I_{t,d}$, considering all chickens present at time t, regardless of the day d on which they were introduced. τ the daily transmission rate through direct contacts, η the relative transmission rate from the environment, and C_t the environmental load at time t, were defined as detailed in (1).

$$\eta = \frac{\zeta}{(1-\zeta)} \frac{1}{\int_{t=0}^{t=H} (1-\Theta)^t dt}$$

$$\tau = \frac{\beta}{N_0 \left[1 + \int_{t=0}^{t=H} (1-\Theta)^t dt\right]}$$

β referred here to the average daily number of effective contacts, i.e. resulting in infection if involving a susceptible and an infected bird, accounting for both direct and environmentally-mediated transmission, in an alternative closed population of which the size N_0 was stable and no chickens left the population. In other words, it was the average daily number of secondary cases caused by an infectious chicken in a fully susceptible and closed population. N_0 was the expected daily maximum number of chickens held at market, $N_0 = N_C(1 + \sum_d s_{\vartheta=24d})$, with N_C being the number of chickens introduced per day, and s_ϑ the probability of a chicken introduced ϑ hours ago being still present at the market. ζ was the proportion of transmission mediated by the environment, and the integral accounted for the decay in infectiousness, at hourly rate $\Theta = 0.043$ (4), of faeces released by infectious chickens. Faeces remained infectious for up to $H = 96$ hours (4).
The prevalence of infection in marketed chickens was the average proportion of infectious chickens over the sampling period T_P, following the daily introduction of chickens into the market (i.e. $\left[\sum_{t=0}^{t=T_P} \sum_d I_{t,d} / \sum_d \left(S_{t,d} + E_{t,d} + I_{t,d}\right)\right] / T_P$). Note that $R_{t,d}$ was ignored. If chickens recovered at the end of their infectious period, this would have led to an overestimation of the prevalence of infection, as $R_{t,d}$ should have been then included in the denominator. However, given the short length of time that chickens spent in markets, the number of recovered/removed chickens was negligible.
The following parameter values were used in the baseline scenario: the length of the infectious period $T_I = 48$ hours, the proportion of transmission mediated by the environment $\zeta = 0.5$, the prevalence of infection among chickens introduced in the market $P_{intr} = 0.005$ (5), the sampling period $T_P = 12$ hours (as chickens were generally introduced when the market opened, and markets remained open all day). The impact of alternative parameter values on the modelled prevalence of infection was assessed through a sensitivity analysis.
The basic reproduction number r was the dominant eigenvalue of the next generation matrix $\mathbf{M}$, for which each element $m_{x,z}$ was the expected number of chickens becoming infected after having spent x hours at the market, and of which the infection was caused by an averaged chicken which became infected after having spent z hours at the market. Each element $m_{x,z}$ was estimated through deterministic simulations: a unique chicken was set as infected after z

hours of presence in the market and we tracked the number of chickens it infected, directly and indirectly (1). Contrary to the stochastic implementation of the model, the number of infected chickens between t and $t + \Delta t$ was $S_{t,d}p_t$, and the number of chickens leaving the market was the product of the number of chickens in a given compartment and the probability of sales. The calculation of r was checked numerically. As shown on Fig. S3, the probability of the viral circulation becoming endemic in a market following the introduction of one infected chicken increased sharply for r>1.

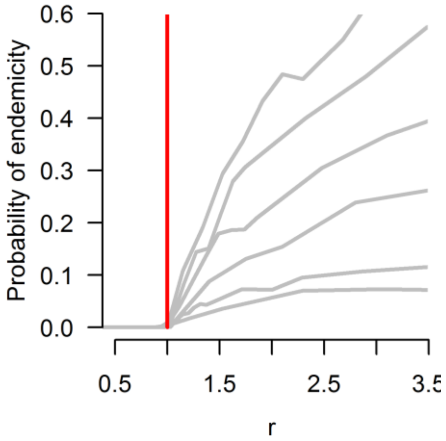

Fig. S3: Probability of a simulation resulting in endemicity within a market as a function of r. Each trajectory corresponds an estimated distribution of the length of time spent by chickens in markets (broilers, sonalis and deshis in Dhaka and Chattogram); at the start of a simulation, a pre-infectious chicken was introduced in the market, and the virus was said to be endemic if at least one marketed chicken was infected after 100 days; as the number of susceptible chickens introduced each day was high (10,000), a viral introduction resulting in a major outbreak in the marketed chicken population would likely lead to disease endemicity, disease invasion being here similar to endemicity.

1.2.5. Mixing of farmed chicken populations in markets.

We assessed the potential of chickens from different types and geographical origins to be sold together in a given market. A farmed chicken population (referred below as chicken population) was defined as all chickens of a given type (i.e. broiler, sonali or deshi) farmed in a given upazila (or district). Networks of broiler, sonali and deshi transactions were reconstructed, but without imputations, in order to avoid over-estimating the level of mixing between chicken populations: the practices of non-interviewed vendors and mobiles traders operating in Dhaka and Chattogram were not imputed, and the origin of chickens sourced from feed dealers was the upazila where the feed dealer's shop was located. Likewise, when chickens were purchased from markets located outside the study area, the origin of chickens was the upazila where the market was located. For each city supplied, we built an adjacency matrix **M** with $m_{ij} = 1$ if two chicken populations were supplied to vendors operating in the same market, and $m_{ij} = 0$ otherwise. We identified the largest clique, which was the largest subset of **M** in which all chicken populations were connected to each other; in other words, the largest subset from which any two chicken populations could be sold together in at least one market.

2. Results

2.1. Description of the practices of study participants.

Tables S3 and S4 provide information on the number of interviewed market vendors and mobile traders, according to the poultry type they sold and their trading practices in Dhaka or Chattogram. In Chattogram, a large majority (91.1%) of interviewed market sold broilers to end-users (i.e. as "retailers"), and a third to half of them sold sonalis, deshis or spent hens to

end-users. In Chattogram, a minority (<5%) of market vendors were wholesalers (market vendors who sold at least one chicken to another trader), unlike in Dhaka where at least a fifth of interviewed market vendors were wholesalers, mostly selling sonalis, deshis or broilers. Around a third (33.5% to 40.5%) of market vendors sold broilers, sonalis or deshis to end-users. In both cities, less than 5% of interviewed market vendors sold cockerels, ducks or other poultry types.

Most interviewed mobile traders who operated in Chattogram traded broilers (75.4%) while a majority of those who operated in Dhaka sold sonalis (52%).

Table S3: Distribution of interviewed market vendors, according to the type of poultry traded. A total of 928 and 461 market vendors operating in Dhaka and Chattogram, respectively, were interviewed during the cross-sectional survey.

	Dhaka		Chattogram	
Poultry type	Retailer[1]	Wholesalers[2]	Retailer[1]	Wholesalers[2]
Broiler	376 (40.5%)	55 (5.9%)	420 (91.1%)	22 (4.8%)
Sonali	311 (33.5%)	180 (19.4%)	146 (31.7%)	12 (2.6%)
Deshi	313 (33.7%)	115 (12.4%)	152 (33%)	9 (2%)
Spent hen	210 (22.6%)	21 (2.3%)	208 (45.1%)	18 (3.9%)
Cockerel	42 (4.5%)	6 (0.6%)	4 (0.9%)	2 (0.4%)
Duck	35 (3.8%)	15 (1.6%)	12 (2.6%)	5 (1.1%)
Other poultry type	39 (4.2%)	17 (1.8%)	16 (3.5%)	3 (0.7%)

[1]Retailers were defined as market vendors who sold all their chickens to end-users. [2]Wholesalers were defined as market vendors who sold at least one chicken to another trader.

Table S4: Distribution of interviewed mobile traders according to the type of poultry they traded. A total of 342 and 179 mobile traders operating in Dhaka and Chattogram, respectively, were interviewed during the cross-sectional survey; one trader operated in both cities.

Poultry type	All (n=520)	Dhaka (n=342)	Chattogram (n=179)
Broiler	188 (36.2%)	53 (15.5%)	135 (75.4%)
Sonali	195 (37.5%)	178 (52%)	17 (9.5%)
Deshi	144 (27.7%)	114 (33.3%)	31 (17.3%)
Spent hen	9 (1.7%)	1 (0.3%)	8 (4.5%)
Cockerel	4 (0.8%)	0 (0%)	4 (2.2%)
Duck	0 (0%)	0 (0%)	0 (0%)
Other poultry type	0 (0%)	0 (0%)	0 (0%)

Tables S5 and S6 show the median numbers of poultry sold by interviewed market vendors and mobile traders, according to poultry type, city, and trading practices (i.e. retailer or wholesaler) and S7 the median proportion of poultry wholesalers sold to other traders, according to poultry type and city. In both cities, wholesalers sold at least twice as many poultry as retailers. In Dhaka city, wholesalers sold more than 80% of their poultry to other traders (except cockerels: 50%) while in Chattogram, wholesalers only sold 30% to 40% of the poultry to other traders.

Table S5: Median number (IQR) of poultry sold by interviewed market vendors over a week according to the type of poultry traded. A total of 928 and 461 market vendors operating in Dhaka and Chattogram, respectively, were interviewed during the cross-sectional survey.

	Dhaka		Chattogram	
Poultry type	Retailer[1]	Wholesaler[2]	Retailer[1]	Wholesaler[2]
Broiler	580<br>(375-775)	2650<br>(1650-5750)	405<br>(360-725)	1525<br>(1169-2138)
Sonali	460<br>(290-800)	3475<br>(1494-9000)	215<br>(101-373)	730<br>(434-810)
Deshi	400<br>(265-600)	1450<br>(900-2550)	210<br>(102-325)	600<br>(450-605)
Spent hen	85<br>(56-129)	700<br>(325-7000)	165<br>(82-255)	625<br>(409-788)
Cockerel	265<br>(108-604)	905<br>(588-1425)	64<br>(56-68)	298<br>(271-324)
Duck	120<br>(41-235)	375<br>(202-762)	80<br>(34-141)	215<br>(178-750)
Other poultry type	100<br>(55-202)	2500<br>(140-4900)	215<br>(128-434)	750<br>(552-1100)

[1]Retailers were defined as market vendors who sold all their chickens to end-users. [2]Wholesalers were defined as vendors who sold at least one chicken to another trader.

Table S6: Median number (IQR) of poultry sold by interviewed mobile traders over a week according to the type of poultry traded. A total of 342 and 179 mobile traders operating in Dhaka and Chattogram, respectively, were interviewed during the cross-sectional survey; one trader operated in both cities.

Poultry type	All (n=520)	Dhaka (n=342)	Chattogram (n=179)
Broiler	7000 (5388-9306)	7000 (6000-11700)	6650 (5265-8212)
Sonali	14000 (7866-18000)	15000 (10000-19262)	5900 (4430-6950)
Deshi	2950 (1294-6081)	4000 (2200-7625)	747 (470-1187)
Spent hen	3500 (930-4450)	3500 (3500-3500)	3565 (816-4738)
Cockerel	3925 (3050-4700)	-	3925 (3050-4700)
Duck	-	-	-
Other poultry type	-	-	-

Table S7: Median (IQR) proportion of poultry sold by market wholesalers to other traders according to the type of poultry traded.

Poultry type	Dhaka	Chattogram
Broiler	80% (70%-100%)	30% (20%-40%)
Sonali	100% (80%-100%)	35% (20%-42.5%)
Deshi	95% (72.5%-100%)	40% (30%-40%)
Spent hen	90% (60%-100%)	30% (20%-40%)
Cockerel	50% (31.2%-72.5%)	40% (35%-45%)
Duck	95% (60%-100%)	40% (40%-50%)
Other poultry type	100% (90%-100%)	40% (30%-50%)

Tables S8 and S9 provide information on the distribution of market vendors and mobile traders according to the number of poultry types traded, and city in which they operated. While some market vendors (1.7%) sold 5 or more poultry types, the majority (90.9%) sold 1 to 3 poultry types. Market vendors in Chattogram were more likely to sell more poultry types than those interviewed in Dhaka markets. Almost all mobile traders (97.1%) sold one poultry type.

Table S8: Distribution of interviewed market vendors according to the number of poultry types traded. Poultry types included: exotic broiler chickens, sonali chickens, deshi chickens, spent hens, cockerels, ducks and other species; a total of 928 and 461 market vendors operating in Dhaka and Chattogram, respectively, were interviewed during the cross-sectional survey.

Number of poultry types	All (n=1389)	Dhaka (n=928)	Chattogram (n=461)
1	486 (35%)	330 (35.6%)	156 (33.8%)
2	587 (42.3%)	451 (48.6%)	136 (29.5%)
3	189 (13.6%)	99 (10.7%)	90 (19.5%)
4	103 (7.4%)	36 (3.9%)	67 (14.5%)
≥5	24 (1.7%)	12 (1.3%)	12 (2.6%)

Table S9: Distribution of interviewed mobile traders according to the number of poultry types traded. Poultry types included: exotic broiler chickens, sonali chickens, deshi chickens, spent hens, cockerels, ducks and other species; a total of 342 and 179 mobile traders operating in Dhaka and Chattogram, respectively, were interviewed during the cross-sectional survey; one trader operated in both cities.

Number of poultry types	All (n=520)	Dhaka (n=342)	Chattogram (n=179)
1	505 (97.1%)	338 (98.8%)	168 (93.9%)
2	11 (2.1%)	4 (1.2%)	7 (3.9%)
3	3 (0.6%)	0 (0%)	3 (1.7%)
4	1 (0.2%)	0 (0%)	1 (0.6%)
≥5	0 (0%)	0 (0%)	0 (0%)

Tables S10 and S11 provide information on the distribution of market vendors operating in Dhaka and Chattogram according to the type of supplier and chicken type. In Dhaka, most interviewed market vendors purchased broilers, sonalis and deshis from a mobile trader or a wholesaler operating in the same market as theirs, or in another market located in the study area. More than 95% of interviewed market vendors who operated in Chattogram purchased broilers, sonalis or deshis from a mobile trader operating in the same market as theirs.

Table S10: Distribution of interviewed market vendors in Dhaka according to their type of supplier. A total of 928 market vendors operating in Dhaka were interviewed during the cross-sectional survey.

Chicken type	Supplier	Retailer[1]	Wholesaler[2]
Broiler	Farmers in a market	0 (0%)	0 (0%)
	Feed dealers[3]	0 (0%)	0 (0%)
	Farm gate	2 (0.5%)	6 (10.9%)
	Vendor's farm	0 (0%)	0 (0%)
	Mobile traders, same market	155 (41.2%)	14 (25.5%)
	Mobile traders, another market, in the study area	87 (23.1%)	2 (3.6%)
	Median number of markets (IQR)	1 (1-2)	1 (1-1)
	Mobile traders, outside of the study area	1 (0.3%)	1 (1.8%)
	Median number of markets (IQR)	2 (2-2)	1 (1-1)
	Wholesalers, same market	19 (5.1%)	4 (7.3%)
	Wholesalers, another market, in the study area	109 (29%)	11 (20%)
	Median number of markets (IQR)	1 (1-1)	1 (1-1)
	Wholesalers, outside of the study area	6 (1.6%)	17 (30.9%)
	Median number of markets (IQR)	1 (1-1)	1 (1-2)
Sonali	Farmers in a market	0 (0%)	0 (0%)
	Feed dealers[3]	0 (0%)	0 (0%)
	Farm gate	0 (0%)	2 (1.1%)
	Vendor's farm	0 (0%)	0 (0%)
	Mobile traders, same market	83 (26.7%)	114 (63.3%)
	Mobile traders, another market, in the study area	47 (15.1%)	7 (3.9%)
	Median number of markets (IQR)	1 (1-1)	1 (1-1)
	Mobile traders, outside of the study area	1 (0.3%)	1 (0.6%)
	Median number of markets (IQR)	2 (2-2)	1 (1-1)
	Wholesalers, same market	75 (24.1%)	21 (11.7%)
	Wholesalers, another market, in the study area	93 (29.9%)	12 (6.7%)
	Median number of markets (IQR)	1 (1-1)	1 (1-1)
	Wholesalers, outside of the study area	14 (4.5%)	26 (14.4%)
	Median number of markets (IQR)	1 (1-1)	1 (1-1)
Deshi	Farmers in a market	0 (0%)	0 (0%)
	Feed dealers[3]	0 (0%)	0 (0%)
	Farm gate	0 (0%)	1 (0.9%)
	Vendor's farm	0 (0%)	0 (0%)
	Mobile traders, same market	93 (29.7%)	59 (51.3%)
	Mobile traders, another market, in the study area	47 (15%)	6 (5.2%)
	Median number of markets (IQR)	1 (1-1)	1 (1-1)
	Mobile traders, outside of the study area	2 (0.6%)	6 (5.2%)
	Median number of markets (IQR)	1.5 (1.3-1.8)	2.5 (1.3-3.8)
	Wholesalers, same market	72 (23%)	22 (19.1%)
	Wholesalers, another market, in the study area	84 (26.8%)	8 (7%)
	Median number of markets (IQR)	1 (1-1)	1 (1-1)
	Wholesalers, outside of the study area	17 (5.4%)	18 (15.7%)
	Median number of markets (IQR)	1 (1-1)	1 (1-1)

[1]Retailers were defined as market vendors who sold all their chickens to end-users. [2]Wholesalers were defined as vendors who sold at least one chicken to another trader. [3]Feed dealer: they supply credit and production inputs (day old chicks, feed) to farmers. They do not purchase or sell poultry but facilitate the trade of poultry between farmers and traders.

Table S11: Distribution of interviewed market vendors in Chattogram according to their type of supplier. A total of 461 market vendors operating Chattogram, were interviewed during the cross-sectional survey.

Chicken type	Supplier	Retailer[1]	Wholesaler[2]
Broiler	Farmers in a market	0 (0%)	0 (0%)
	Feed dealers[3]	0 (0%)	0 (0%)
	Farm gate	2 (0.5%)	0 (0%)
	Vendor's farm	0 (0%)	0 (0%)
	Mobile traders, same market	413 (98.3%)	22 (100%)
	Mobile traders, another market, in the study area	2 (0.5%)	0 (0%)
	Median number of markets (IQR)	1 (1-1)	-
	Mobile traders, outside of the study area	0 (0%)	0 (0%)
	Median number of markets (IQR)	-	-
	Wholesalers, same market	8 (1.9%)	0 (0%)
	Wholesalers, another market, in the study area	1 (0.2%)	0 (0%)
	Median number of markets (IQR)	1 (1-1)	-
	Wholesalers, outside of the study area	0 (0%)	0 (0%)
	Median number of markets (IQR)	-	-
Sonali	Farmers in a market	0 (0%)	0 (0%)
	Feed dealers[3]	0 (0%)	0 (0%)
	Farm gate	0 (0%)	0 (0%)
	Vendor's farm	0 (0%)	0 (0%)
	Mobile traders, same market	140 (95.9%)	12 (100%)
	Mobile traders, another market, in the study area	0 (0%)	0 (0%)
	Median number of markets (IQR)	-	-
	Mobile traders, outside of the study area	0 (0%)	0 (0%)
	Median number of markets (IQR)	-	-
	Wholesalers, same market	6 (4.1%)	0 (0%)
	Wholesalers, another market, in the study area	1 (0.7%)	0 (0%)
	Median number of markets (IQR)	1 (1-1)	-
	Wholesalers, outside of the study area	0 (0%)	0 (0%)
	Median number of markets (IQR)	-	-
Deshi	Farmers in a market	0 (0%)	0 (0%)
	Feed dealers[3]	0 (0%)	0 (0%)
	Farm gate	0 (0%)	0 (0%)
	Vendor's farm	0 (0%)	0 (0%)
	Mobile traders, same market	146 (96.1%)	9 (100%)
	Mobile traders, another market, in the study area	1 (0.7%)	0 (0%)
	Median number of markets (IQR)	1 (1-1)	-
	Mobile traders, outside of the study area	0 (0%)	0 (0%)
	Median number of markets (IQR)	-	-
	Wholesalers, same market	6 (3.9%)	0 (0%)
	Wholesalers, another market, in the study area	0 (0%)	0 (0%)
	Median number of markets (IQR)	-	-
	Wholesalers, outside of the study area	0 (0%)	0 (0%)
	Median number of markets (IQR)	-	-

[1]Retailers were defined as market vendors who sold all their chickens to end-users. [2]Wholesalers were defined as vendors who sold at least one chicken to another trader. [3]Feed dealer: they supply credit and production inputs (day old chicks, feed) to farmers. They do not purchase or sell poultry but facilitate the trade of poultry between farmers and traders.

Tables S12 and S13 provide information on the market vendors' trading practices that impacted the length of time chickens stayed in markets. In both Dhaka and Chattogram, vendors reported surplus to be frequent. The median proportion of chickens left unsold ranged between 13%-26% according to the type of chicken. Wholesalers' practices were similar to retailers', except wholesalers who sold sonalis or deshis in Dhaka. Indeed, they had a lower frequency of surplus

(33.3%, 42.9%, respectively), and less poultry left unsold when experiencing a surplus (7.9% and 9.5%).

Table S12: Trading practices of market vendors in Dhaka. The focus is on practices impacting on the length of time during which chickens remained in markets; a total of 928 market vendors operating in Dhaka were interviewed during the cross-sectional survey; a surplus is defined as chickens left unsold when receiving a new supply of chickens.

		Retailer[1]	Wholesaler[2]
Broiler	n	376	55
	n (%) surplus at least once[3]	364 (96.8%)	43 (78.2%)
	Median frequency of surplus (IQR)[4]	100% (71.4%-100%)	100% (22.6%-100%)
	Proportion of unsold chickens, median (IQR)[5]	13.6% (8.8%-21.2%)	5.7% (4.3%-7.4%)
	n (%), prioritise surplus[6]	176 (48.4%)	34 (79.1%)
	n (%), supplied with chickens everyday	312 (83%)	53 (96.4%)
Sonali	n	311	180
	n (%) surplus at least once[3]	306 (98.4%)	114 (63.3%)
	Median frequency of surplus (IQR)[4]	100% (85.7%-100%)	33.3% (0%-100%)
	Proportion of unsold chickens, median (IQR)[5]	15.8% (10.3%-27.4%)	7.9% (5.7%-18%)
	n (%), prioritise surplus[6]	152 (49.7%)	45 (39.5%)
	n (%), supplied with chickens everyday	245 (78.8%)	173 (96.1%)
Deshi	n	313	115
	n (%) surplus at least once[3]	307 (98.1%)	67 (58.3%)
	Median frequency of surplus (IQR)[4]	100% (75%-100%)	42.9% (0%-100%)
	Proportion of unsold chickens, median (IQR)[5]	17.1% (9.7%-28.7%)	9.5% (6.8%-24.6%)
	n (%), prioritise surplus[6]	145 (47.2%)	37 (55.2%)
	n (%), supplied with chickens everyday	240 (76.7%)	111 (96.5%)

[1]Retailers were defined as market vendors who sold all their chickens to end-users. [2]Wholesalers were defined as vendors who sold at least one chicken to another trader. [3]Within the last 7 days the vendors offered chickens for sale. [4]Only vendors who reported surplus are considered. [5]When having chickens left unsold at the end of the day. [6]When chickens are left unsold, their sale is then prioritised over the newly supplied chickens.

Table S13: Trading practices of market vendors in Chattogram. The focus is on practices impacting on the length of time during which chickens remained in markets; a total of 461 market vendors operating in Chattogram were interviewed during the cross-sectional survey; a surplus is defined as chickens left unsold when receiving a new supply of chickens.

		Retailer[1]	Wholesaler[2]
Broiler	n	420	22
	n (%) surplus at least once[3]	415 (98.8%)	22 (100%)
	Median frequency of surplus (IQR)[4]	100% (71.4%-100%)	100% (57.1%-100%)
	Proportion of unsold chickens, median (IQR)[5]	16.8% (11.2%-24.9%)	7.1% (6%-9.6%)
	n (%), prioritise surplus[6]	57 (13.7%)	4 (18.2%)
	n (%), supplied with chickens everyday	398 (94.8%)	21 (95.5%)
Sonali	n	146	12
	n (%) surplus at least once[3]	138 (94.5%)	12 (100%)
	Median frequency of surplus (IQR)[4]	71.4% (42.9%-100%)	100% (64.3%-100%)
	Proportion of unsold chickens, median (IQR)[5]	24.7% (13.7%-43.8%)	8.5% (5.6%-25%)
	n (%), prioritise surplus[6]	26 (18.8%)	1 (8.3%)
	n (%), supplied with chickens everyday	117 (80.1%)	12 (100%)
Deshi	n	152	9
	n (%) surplus at least once[3]	148 (97.4%)	8 (88.9%)
	Median frequency of surplus (IQR)[4]	71.4% (42.9%-100%)	100% (42.9%-100%)
	Proportion of unsold chickens, median (IQR)[5]	25.9% (13.9%-45.8%)	16.4% (6.9%-36.5%)
	n (%), prioritise surplus[6]	26 (17.6%)	0 (0%)
	n (%), supplied with chickens everyday	120 (78.9%)	9 (100%)

[1]Retailers were defined as market vendors who sold all their chickens to end-users. [2]Wholesalers were defined as vendors who sold at least one chicken to another trader. [3]Within the last 7 days the vendors offered chickens for sale. [4]Only vendors who reported surplus are considered. [5]When having chickens left unsold at the end of the day. [6]When chickens are left unsold, their sale is then prioritised over the newly supplied chickens.

Table S14 provides information on the distribution of mobile traders operating in Dhaka and Chattogram according to the type of supplier and chicken type, and table S15 presents the distribution of mobile traders according to the number of markets and the city they operated in. Most mobile traders purchased broilers from a feed-dealer (81.4%), sonalis from a feed dealer or directly form a farm (84.1) and deshis from mobile traders operating outside the study area (70.1%). Most interviewed mobile traders who operated in Dhaka sold poultry at a single market (58.5%, 50.6%, 77.2% for broilers, sonalis and deshis respectively) whereas in Chattogram, mobile traders who sold broilers or sonalis mostly operated in 5 or 6 markets while those who sold deshis mostly operated in 1 to 3 markets.

Table S14: Distribution of interviewed mobile traders according to their type of supplier. A total of 342 and 179 mobile traders operating in Dhaka and Chattogram, respectively, were interviewed during the cross-sectional survey; one trader operated in both cities.

Chicken type	Supplier	All	Dhaka	Chattogram
Broiler	Farmers in a market	3 (1.6%)	3 (5.7%)	0 (0%)
	Feed dealers[1]	153 (81.4%)	34 (64.2%)	119 (88.1%)
	Farm gate	29 (15.4%)	11 (20.8%)	18 (13.3%)
	Mobile trader's farm	15 (8%)	0 (0%)	15 (11.1%)
	Mobile traders, in the study area	2 (1.1%)	0 (0%)	2 (1.5%)
	Median number of markets (IQR)	1 (1-1)	-	1 (1-1)
	Mobile traders, outside of the study area	13 (6.9%)	8 (15.1%)	5 (3.7%)
	Median number of markets (IQR)	1 (1-1)	1 (1-1)	1 (1-1)
	Wholesalers, in the study area	0 (0%)	0 (0%)	0 (0%)
	Median number of markets (IQR)	-	-	-
	Wholesalers, outside of the study area	8 (4.3%)	8 (15.1%)	0 (0%)
	Median number of markets (IQR)	1 (1-1)	1 (1-1)	-
Sonali	Farmers in a market	5 (2.6%)	5 (2.8%)	0 (0%)
	Feed dealers[1]	92 (47.2%)	75 (42.1%)	17 (100%)
	Farm gate	72 (36.9%)	72 (40.4%)	0 (0%)
	Mobile trader's farm	1 (0.5%)	0 (0%)	1 (5.9%)
	Mobile traders, in the study area	0 (0%)	0 (0%)	0 (0%)
	Median number of markets (IQR)	-	-	-
	Mobile traders, outside of the study area	31 (15.9%)	31 (17.4%)	0 (0%)
	Median number of markets (IQR)	1 (1-2)	1 (1-2)	NA
	Wholesalers, in the study area	0 (0%)	0 (0%)	0 (0%)
	Median number of markets (IQR)	-	-	-
	Wholesalers, outside of the study area	16 (8.2%)	16 (9%)	0 (0%)
	Median number of markets (IQR)	1 (1-1)	1 (1-1)	-
Deshi	Farmers in a market	22 (15.3%)	12 (10.5%)	11 (35.5%)
	Feed dealers[1]	17 (11.8%)	16 (14%)	1 (3.2%)
	Farm gate	25 (17.4%)	11 (9.6%)	15 (48.4%)
	Mobile trader's farm	0 (0%)	0 (0%)	0 (0%)
	Mobile traders, in the study area	0 (0%)	0 (0%)	0 (0%)
	Median number of markets (IQR)	-	-	-
	Mobile traders, outside of the study area	101 (70.1%)	86 (75.4%)	15 (48.4%)
	Median number of markets (IQR)	3 (2-3)	3 (2-3)	3 (1.5-3)
	Wholesalers, in the study area	0 (0%)	0 (0%)	0 (0%)
	Median number of markets (IQR)	-	-	-
	Wholesalers, outside of the study area	10 (6.9%)	9 (7.9%)	1 (3.2%)
	Median number of markets (IQR)	1 (1-1.75)	1 (1-1)	2 (2-2)

[1]Feed dealer: they supply credit and production inputs (day old chicks, feed) to farmers. They do not purchase or sell poultry, but facilitate the trade of poultry between farmers and traders.

Table S15: Distribution of interviewed mobile traders according to the number of markets where they sold chickens, according to chicken type. A total of 342 and 179 mobile traders operating in Dhaka and Chattogram, respectively, were interviewed during the cross-sectional survey.

Chicken type	Number of markets	n (%), Dhaka	n (%), Chattogram
Broiler	1	31 (58.5%)	4 (3%)
	2	17 (32.1%)	8 (5.9%)
	3	4 (7.5%)	13 (9.6%)
	4	1 (1.9%)	21 (15.6%)
	5	0 (0%)	26 (19.3%)
	6	0 (0%)	55 (40.7%)
	7	0 (0%)	7 (5.2%)
	8	0 (0%)	1 (0.7%)
	≥9	0 (0%)	0 (0%)
Sonali	1	90 (50.6%)	0 (0%)
	2	57 (32%)	1 (5.9%)
	3	27 (15.2%)	1 (5.9%)
	4	4 (2.2%)	1 (5.9%)
	5	0 (0%)	1 (5.9%)
	6	0 (0%)	11 (64.7%)
	7	0 (0%)	2 (11.8%)
	≥8	0 (0%)	0 (0%)
Deshi	1	88 (77.2%)	9 (29%)
	2	20 (17.5%)	7 (22.6%)
	3	6 (5.3%)	4 (12.9%)
	4	0 (0%)	5 (16.1%)
	5	0 (0%)	1 (3.2%)
	6	0 (0%)	5 (16.1%)
	≥7	0 (0%)	0 (0%)

Table S16 presents information on the prices at which chicken types were sold according to city and whether they were sold to another trader (i.e. as wholesale) or not. Broilers were sold at the same median price in Dhaka and Chattogram (110 BDT), but the median price of sonalis and deshis was 20% lower in Dhaka than in Chattogram

Table S16: Prices of chickens sold by market vendors. Prices are expressed in Bangladesh takas. Median values and interquartile ranges (IQR) are shown according to poultry type, city and transaction (i.e. retail vs wholesale).

	Chattogram		Dhaka	
	Retail price median (IQR)	Wholesale price median (IQR)	Retail price median (IQR)	Wholesale price median (IQR)
Broiler	115 (110-130)	110 (106-120)	120 (110-130)	110 (106-124)
Sonali	225 (210-250)	210 (200-220)	180 (150-200)	164 (140-189)
Deshi	310 (300-320)	300 (300-315)	250 (220-300)	242 (230-260)

Tables S17-S19 present the results of the longitudinal study. All but one market vendor trading broilers sold this type of chicken every month, contrary to sonalis and deshis which were sold intermittently by several vendors. Overall, chicken sales tended to decrease during the rainy season (June-August). Indeed, vendors were less likely to offer deshis for sales from July to September, and the number of deshis sold by vendors offering these for sale decreased in June. Likewise, the sales of broilers and sonalis dropped at their lowest from June to August.

Table S17: Number of upazilas and districts visited by mobile traders, longitudinal study. Interview-period: the 4 days a mobile trader was interviewed per month.

	Broiler	Sonali	Deshi
No. of traders	18	18	19
No. of upazilas/trader/interview-period, mean	2	2.1	2.2
No. of upazilas/trader/year, mean (range)	7.5 (1-17)	8.8 (1-25)	8 (1-24)
No. of districts/trader/interview-period, mean	1.3	1.8	1.7
No. of districts /trader/year, mean (range)	2.4 (1-5)	5.1 (1-14)	4.5 (1-11)

Table S18: Results of logistic regression models with the sales of chickens as an outcome variable, longitudinal study. Models were only used for broilers, sonalis and deshis given the small number of interviewed market vendors selling ducks, and that all but one vendors selling broilers sold this type of chicken every month. OR (95% CI), p: odds ratio (95% confidence interval), p-value.

	Broiler	Sonali	Deshi	Duck
Sell, at least once	19 (44.2%)	32 (74.4%)	24 (55.8%)	9 (20.9%)
Sell, every month	18 (41.9%)	25 (58.1%)	14 (32.6%)	3 (7%)
Sell, intermittently	1 (2.3%)	7 (16.3%)	10 (23.3%)	6 (14%)
Month	OR (95% CI), p	OR (95% CI), p	OR (95% CI), p	
January	-	Reference	Reference	-
February	-	1.00 (0.07,14.05), p=1	1.00 (0.01,97.19), p=1	-
March	-	0.07 (0.00,2.41), p=0.14	1.00 (0.01,97.19), p=1	-
April	-	2.27 (0.18,28.67), p=0.53	1.00 (0.01,97.19), p=1	-
May	-	2.27 (0.18,28.67), p=0.53	5.54 (0.11,288), p=0.4	-
June	-	1.00 (0.07,14.05), p=1	0.03 (0.00,1.43), p=0.08	-
July	-	1.00 (0.07,14.05), p=1	0.02 (0.00,0.82), p=0.04	-
August	-	2.27 (0.18,28.67), p=0.53	0.02 (0.00,0.82), p=0.04	-
September	-	0.07 (0.00,2.41), p=0.14	0.02 (0.00,0.82), p=0.04	-
October	-	0.35 (0.02,6.36), p=0.47	0.03 (0.00,1.43), p=0.08	-
November	-	0.07 (0.00,2.41), p=0.14	0.06 (0.00,2.95), p=0.16	-
December	-	2.27 (0.18,28.67), p=0.53	1.00 (0.01,97.19), p=1	-

Table S19: Results of linear regression models with the number of sold chickens as an outcome variable, longitudinal study. The number of chickens was log-transformed; coef (95% CI), p: coefficient (95% confidence interval), p-value.

	Broiler	Sonali	Deshi
Sell, at least once	19 (44.2%)	32 (74.4%)	24 (55.8%)
Sell, every month	18 (41.9%)	25 (58.1%)	14 (32.6%)
Sell, intermittently	1 (2.3%)	7 (16.3%)	10 (23.3%)
Month	coef (95% CI), p	coef (95% CI), p	coef (95% CI), p
January	Reference	Reference	Reference
February	-0.05 (-0.24,0.13), p=0.57	0.01 (-0.16,0.19), p=0.87	-0.13 (-0.41,0.14), p=0.35
March	0.00 (-0.19,0.18), p=0.97	-0.06 (-0.24,0.12), p=0.53	-0.15 (-0.43,0.12), p=0.28
April	-0.14 (-0.32,0.05), p=0.15	-0.12 (-0.29,0.06), p=0.21	-0.18 (-0.46,0.09), p=0.2
May	-0.05 (-0.23,0.14), p=0.6	0.08 (-0.10,0.26), p=0.38	-0.05 (-0.32,0.23), p=0.74
June	-0.19 (-0.38,-0.01), p=0.04	-0.35 (-0.53,-0.17), p<0.01	-0.53 (-0.82,-0.24), p<0.01
July	-0.20 (-0.39,-0.02), p=0.03	-0.05 (-0.23,0.13), p=0.6	-0.06 (-0.35,0.23), p=0.69
August	-0.25 (-0.43,-0.06), p=0.01	-0.32 (-0.5,-0.15), p<0.01	-0.06 (-0.35,0.23), p=0.68
September	-0.11 (-0.30,0.07), p=0.23	0.08 (-0.11,0.27), p=0.4	-0.18 (-0.47,0.11), p=0.22
October	0.00 (-0.19,0.18), p=1	0.15 (-0.03,0.33), p=0.11	-0.26 (-0.55,0.02), p=0.07
November	0.05 (-0.13,0.23), p=0.59	0.02 (-0.16,0.21), p=0.81	-0.10 (-0.39,0.18), p=0.47
December	0.04 (-0.14,0.22), p=0.68	0.23 (0.06,0.41), p=0.01	-0.12 (-0.4,0.16), p=0.41

2.2. Sequences of actors.

Table S20 shows the median number of poultry sold through market vendors in each city over a week. In Dhaka, these estimations were conducted by imputing, or not, sales in markets where vendors were not interviewed. Although the number of poultry sold within a week, and the

proportion of sales to end-users increased with imputations, the proportion of sales accounted by each poultry type remained stable.

In Chattogram, all poultry types were mostly sold to end-users (>79.1%) and broilers were the main poultry type traded through market vendors (62.9%). In contrast, in Dhaka, sonalis were the main poultry type traded (65.3%, no imputations), and most sonalis were sold to other traders (87.1%, no imputations). This pattern was driven by one large market through which most (57.7%, 2.5th-97.5th quantile range: 49.5%-58.9%) sonalis sold in Dhaka transited. If only retail sales were considered, broilers were the main poultry type traded in Dhaka (35.1%, no imputations), followed by sonalis (30.9%, no imputations), and deshis (24.6%, no imputations). The proportion of retail sales accounted for by sonalis and deshis remained greater in Dhaka than in Chattogram (11.2% and 10.0%, respectively).

Table S20: Estimated number of poultry sold through market vendors over a week in Dhaka and Chattogram. 95%I: the interval defined by the 2.5% and 97.5% quantiles; proportion: the proportion of chickens sold, for each type; Imputations: sales in markets where vendors were not interviewed were simulated; end-users refer to consumers and restaurateurs, other customers are traders who then sell chickens outside markets.

Poultry type	n median (95%I)	Proportion median (95%I)	Proportion to end-users median (95%I)
Dhaka, imputations			
Broiler	673,047 (632,354-720,343)	18.3% (16.7%-19.6%)	72.6% (67.9%-77.3%)
Sonali	2,184,339 (2,131,006-2,530,449)	60.6% (59%-63.8%)	17% (14.6%-18.4%)
Deshi	494,253 (459,135-536,000)	13.5% (12.1%-14.6%)	61.4% (55.6%-64.8%)
Spent hen	124,589 (121,345-128,890)	3.4% (3.1%-3.6%)	41.7% (39.9%-43.4%)
Cockerel	49,473 (42,599-58,349)	1.3% (1.1%-1.6%)	89.2% (87.8%-90.9%)
Duck	19,951 (19,496-20,514)	0.5% (0.5%-0.6%)	55.3% (53.8%-56.8%)
Other	78,929 (78,059-79,866)	2.2% (2%-2.2%)	27.2% (26%-28.7%)
Dhaka, no imputations			
Poultry type	n median (95%I)	Proportion median (95%I)	Proportion to end-users median (95%I)
Broiler	470,981 (468,662-473,434)	14.9% (14.8%-15%)	64.3% (64%-64.6%)
Sonali	2,066,869 (2,046,577-2,087,928)	65.3% (65%-65.5%)	12.9% (12.7%-13%)
Deshi	404,526 (401,824-407,256)	12.8% (12.7%-12.9%)	52.6% (52.2%-53%)
Spent hen	102,249 (101,795-102,721)	3.2% (3.2%-3.3%)	30.1% (29.8%-30.4%)
Cockerel	25,497 (23,665-28,040)	0.8% (0.7%-0.9%)	86% (84.9%-87.3%)
Duck	18,813 (18,538-19,085)	0.6% (0.6%-0.6%)	52.4% (51.3%-53.4%)
Other	77,660 (77,309-78,007)	2.5% (2.4%-2.5%)	23.8% (23.6%-24%)
Chattogram			
Poultry type	n median (95%I)	Proportion median (95%I)	Proportion to end-users median (95%I)
Broiler	275,597 (274,412-276,860)	62.9% (61.9%-63.6%)	95.7% (95.7%-95.8%)
Sonali	48,922 (46,899-53,659)	11.2% (10.7%-12.2%)	96.5% (96.1%-96.9%)
Deshi	44,030 (41,294-49,793)	10% (9.5%-11.2%)	96.9% (96.6%-97.3%)
Spent hen	57,671 (56,775-59,374)	13.2% (12.9%-13.6%)	94.5% (94.4%-94.7%)
Cockerel	1596 (1484-1739)	0.4% (0.3%-0.4%)	84.4% (83.3%-85.7%)
Duck	3022 (2616-3423)	0.7% (0.6%-0.8%)	79.1% (75.9%-81.7%)
Other	6487 (6345-6639)	1.5% (1.4%-1.5%)	91.1% (90.6%-91.7%)

Table S21 presents the proportion of chickens supplied by each type of network origin (i.e. source node), for each chicken type and city, when the transaction networks were reconstructed without imputations. In Dhaka and Chattogram, most deshis were purchased from mobile traders operating outside the study area. Feed dealers facilitated the purchases and sales of most broilers and sonalis sold in Chattogram. In Dhaka, a quarter of broilers were sourced from feed dealers, a quarter from mobile traders operating in the study area, and another quarter from wholesalers operating outside of the study area. Most sonalis sold in Dhaka were directly sourced from farms (50.2%).

Table S21: Estimated proportion of chickens supplied by each type of network origin (i.e. source node) without imputations. Non-visited markets were ignored; if interviewed market vendors and mobile traders reported purchasing chickens from wholesalers and/or mobile traders in markets where such actors were not interviewed, the trading practices of those non-interviewed actors were not imputed.

Dhaka			
Type of network origin	Broiler	Sonali	Deshi
Farm	10.8% (10.7%-10.8%)	50.2% (50%-50.3%)	7.7% (7.6%-7.9%)
Feed dealer	23.7% (23.5%-23.8%)	30.9% (30.6%-31.1%)	18.7% (18.5%-19%)
Mobile trader, study area[1]	27.6% (27.4%-27.8%)	1.6% (1.6%-1.6%)	14% (13.8%-14.2%)
Wholesaler, study area[1]	5.1% (4.9%-5.3%)	0.4% (0.3%-0.4%)	1.9% (1.9%-2%)
Mobile trader, outside[2]	7.1% (7%-7.1%)	10.4% (10.3%-10.5%)	42.9% (42.6%-43.2%)
Wholesaler, outside[2]	25.8% (25.5%-26.1%)	6.6% (6.6%-6.7%)	14.6% (14.5%-14.8%)
Chattogram			
Type of network origin	Broiler	Sonali	Deshi
Farm	9.7% (9.4%-10.1%)	0.4% (0.4%-0.5%)	17.1% (15.1%-18.8%)
Feed dealer	82.8% (82.4%-83.1%)	92% (91.6%-92.7%)	1.9% (1.7%-2.4%)
Mobile trader, study area[1]	5.6% (5.5%-5.6%)	6.6% (6%-7%)	28.2% (25.5%-30.1%)
Wholesaler, study area[1]	0.6% (0.6%-0.6%)	1% (0.8%-1.1%)	1.2% (1.1%-1.3%)
Mobile trader, outside[2]	1.3% (1.3%-1.4%)	0% (0%-0%)	49.6% (46.3%-54.3%)
Wholesaler, outside[2]	0% (0%-0%)	0% (0%-0%)	1.8% (1.6%-2%)

[1]Non-interviewed actors operating in markets inside the study area. [2]Non-interviewed actors operating in markets outside the study area.

2.3. Market catchment areas.

Table S22 shows the estimated spatial distribution of chickens sourced by feed dealers according to the study considered. Results were consistent between the longitudinal and cross-sectional studies. In the main manuscript, we report results using feed dealers' practices estimated through the longitudinal study (broilers and sonalis), and data gathered during the additional cross-sectional study which exclusively investigated deshi and duck trading patterns, which were not fully captured by the cross-sectional and longitudinal studies (deshis). Indeed, during the cross-sectional study only 5 interviewed feed dealers sold deshis (Table S22), while during the additional study focused on duck and deshi trade, 31 mobile traders trading deshis were interviewed. These mobile traders declared purchasing similar proportions of deshis in the same upazila, the same district (other upazilas) and other districts as their offices (0.68; 0.26;0.06), than feed dealers interviewed during the cross-sectional study (Table S22).

Table S22: Estimated distribution of chickens sourced by feed dealers according to chicken type and the study through which feed dealers were interviewed. Same upazila: proportion of chickens sourced from the same upazila as the feed dealers' headquarter; all, DHA, CTG: feed dealers supplying both cities, Dhaka, Chattogram.

		Cross-sectional study			Longitudinal study		
		all	DHA	CTG	all	DHA	CTG
Broiler	n (feed dealers)	47	23	24	16	7	9
	Same upazila	54.9%	39%	67%	52.6%	41.0%	61.7%
	Same district (other upazilas)	30.3%	35.8%	26.1%	44.7%	56.1%	35.8%
	Other districts	14.8%	25.2%	7%	2.7%	2.9%	2.6%
Sonali	n (feed dealers)	36	28	8	17	8	9
	Same upazila	36.7%	34.4%	45.0%	36.7%	20.5%	51.0%
	Same district (other upazilas)	42.3%	43.9%	36.7%	59.4%	78.7%	42.2%
	Other districts	21.0%	21.7%	18.3%	4.0%	0.8%	6.8%
Deshis	n (feed dealers)	5	4	1	-	-	-
	Same upazila	60%	68%	20%	-	-	-
	Same district (other upazilas)	20%	12%	60%	-	-	-
	Other districts	20%	20%	20%			

Figures S4 and S5 shows the estimated catchment areas of broilers and sonalis sold in Dhaka and Chattogram markets under different sets of assumptions about feed dealers' practices. Catchment areas varied little when using the longitudinal or the cross-sectional studies to inform feed dealers' practices. The upazilas supplying the greatest proportions of broilers and sonalis to Dhaka and Chattogram markets remained unchanged.

Fig. S6 shows the hierarchical clustering of Dhaka and Chattogram markets based on the similarity of their catchment areas for broilers, sonalis and deshis. Most markets' catchment areas were similar, or substantially overlapped, with the catchment areas of other markets in the same city. This suggested that, for each chicken type, multiple markets in a city sold chickens sourced from the same geographical areas.
Pianka's indexes calculated to compare catchment areas generated using baseline data (used for main text results) with catchment areas generated using other data sources (shown here) were >0.99, regardless of city or poultry type.

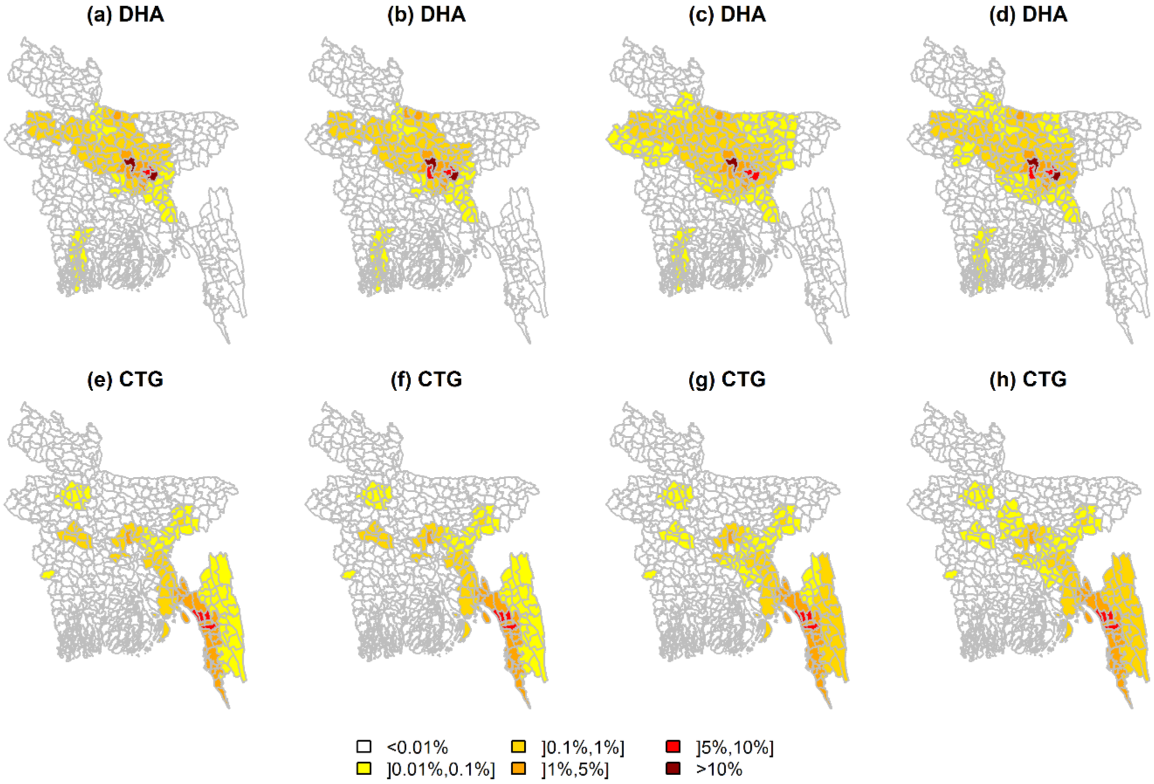

Fig. S4: Origins of broilers sold in Dhaka and Chattogram markets, under different sets of assumptions. Catchment areas of Dhaka (a-d) and Chattogram (e-h) are presented based on data collected exclusively in Dhaka (a) or Chattogram (e) during the longitudinal study, across the study area during the longitudinal study (b, f), exclusively in Dhaka or Chattogram during the cross-sectional study (c, g), and across the study area during the cross-sectional study (d, h).

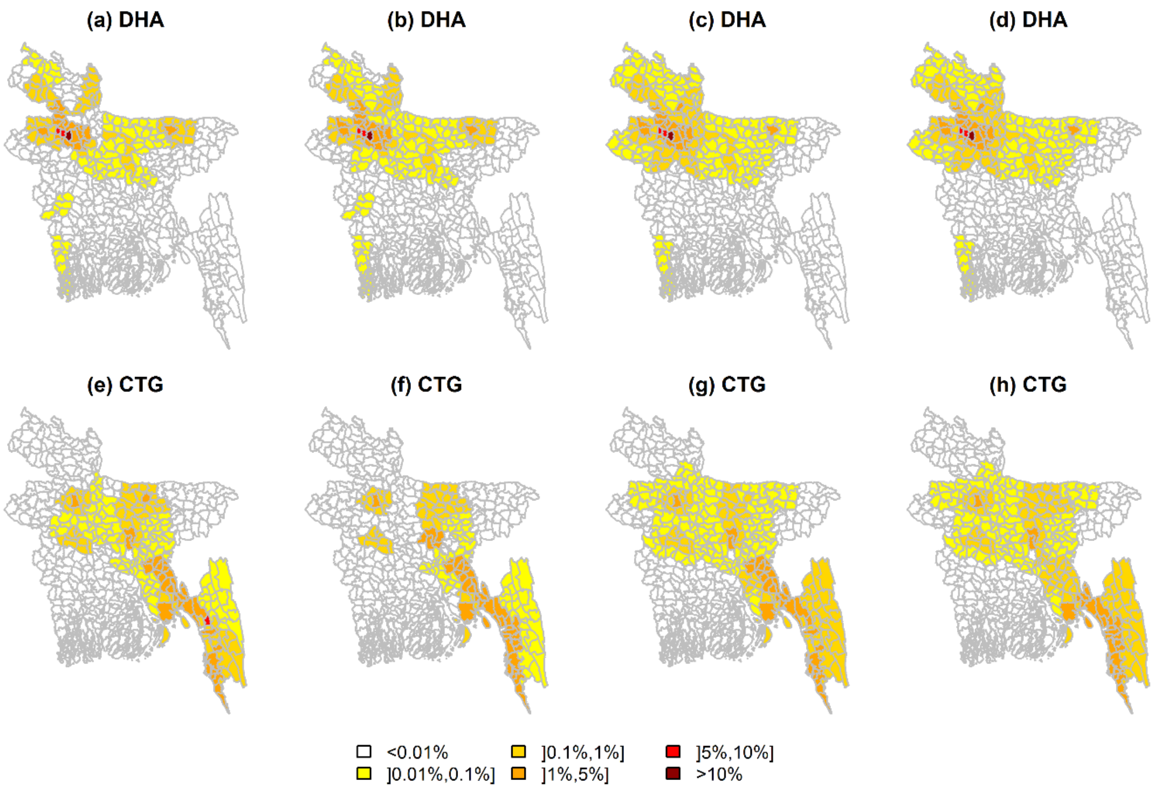

Fig. S5: Origins of sonalis sold in Dhaka and Chattogram markets, under different sets of assumptions. Catchment areas of Dhaka (a-d) and Chattogram (e-h) are presented based on data collected exclusively in Dhaka (a) or Chattogram (e) during the longitudinal study, across the study area during the longitudinal study (b, f), exclusively in Dhaka or Chattogram during the cross-sectional study (c, g), and across the study area during the cross-sectional study (d, h).

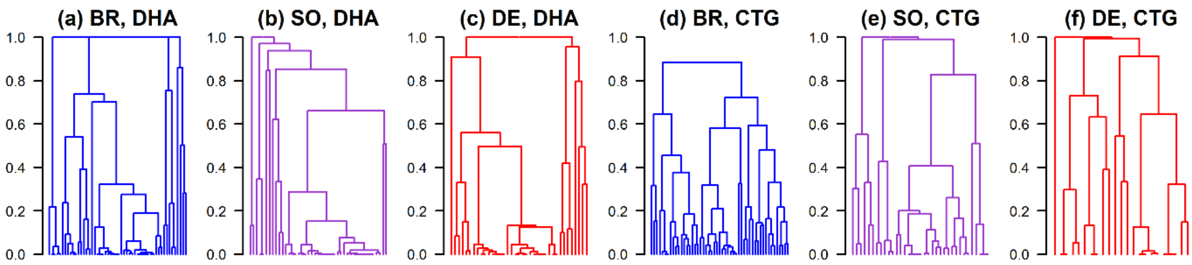

Fig. S6: Hierarchical clustering of Dhaka (DHA) and Chattogram (CTG) markets based on the similarity of their catchment areas for broilers (BR), sonalis (SO) and deshis (DE); the distance between any two markets (y-axis) was 1-[Pianka index], with a value of 0 meaning that two markets had similar catchment areas, and a value of 1 that those areas did not overlap.

2.4. Mixing of farmed chicken populations in markets.

With district or upazila as the population unit, the largest subset of chicken populations in which any two populations could be sold together in at least one market encompassed ≥77.5% of chicken populations supplying a city, and ≥97.9% of a city catchment area (Table S23). Chickens from ≥84.2% of populations could mix in at least one market with chickens from ≥80% of populations.

Table S23: Mixing of farmed chicken populations in markets.

Supplied city	Chattogram	Dhaka	Chattogram	Dhaka
Population unit	Upazila	Upazila	District	District
Number of farmed chicken populations	235	423	40	57
Largest clique				
Number of populations (%)	215 (91.5%)	328 (77.5%)	35 (87.5%)	45 (78.9%)
Cumulative catchment area	97.9%	98.2%	99.4%	99.0%
Number of populations (%) in contact, through markets, with				
≥80% of populations	233 (99.1%)	356 (84.2%)	38 (95%)	48 (84.2%)
≥90% of populations	222 (94.5%)	225 (53.2%)	33 (82.5%)	35 (61.4%)
≥95% of populations	196 (83.4%)	167 (39.5%)	30 (75%)	28 (49.1%)
≥99% of populations	190 (80.9%)	47 (11.1%)	8 (20%)	1 (1.8%)
Cumulative catchment area of populations in contact through markets, with other populations accounting for				
≥80% of the catchment area	99.9%	99.9%	100%	99.9%
≥90% of the catchment area	99.9%	99.9%	99.9%	99.9%
≥95% of the catchment area	99.9%	99.7%	99.9%	99.7%
≥99% of the catchment area	98.8%	98.9%	99.8%	99.0%

2.5. Movements of chickens and traders between markets within cities.

Table S24 summarises information on the networks of markets shaped by the transaction of chickens between markets, while table S25 presents the same information for networks shaped by movement of traders between markets. In each table, the information is presented for each city. In Dhaka, the network was mainly shaped by transactions of chickens between markets, unlike in Chattogram where the network was mainly shaped by movements of traders between markets.

Table S24: Networks of markets shaped by the transaction of chickens between markets. Node: visited market; edge: transaction of chickens (broilers, sonalis and deshis) between markets; networks were unweighted and directed.

	Chattogram	Dhaka
no. of nodes	55	64
no. of isolated nodes	47 (85.5%)	17 (26.6%)
no. of connected nodes	8 (14.5%)	47 (73.4%)
Size of the largest weak component	5 (62.5%)[1]	47 (100%)[1]
Size of the largest strong component	2 (25%)	2 (4.3%)
no. of edges	7	83
density	0.002	0.02
no. of reciprocated edges	2	2
no. of origins (i.e. in-degree=0; out-degree>0)	1 (12.5%)	4 (8.5%)
no. of intermediary nodes (i.e. in-degree>0; out-degree>0)	2 (25%)	8 (17%)
no. of sinks (i.e. in-degree>0; out-degree=0)	5 (62.5%)	35 (74.5%)
sinks: average in-degree (max)	1 (1)	1.9 (4)
No. of edges originating from the 2 main city markets	-	53 (63.9%)

[1]Here and below, the denominator was the number of connected nodes

Table S25: Networks of markets shaped by the movements of traders between markets. Node: visited market; edge: an edge was formed if two markets were visited by the same trader; networks were unweighted and undirected; clustering coefficient and average path length were compared to the same metrics for 1000 random networks with the same number of nodes and edges, with the p-value being the proportion of simulated metrics equal or higher than the observed ones.

	Chattogram	Dhaka
no. of nodes	55	64
no. of isolated nodes	4 (7.3%)	13 (20.3%)
no. of connected nodes	51 (92.7%)	51 (79.7%)
Size of the largest weak component	51 (100%)[1]	51 (100%)[1]
no. of edges	488	121
density	0.33	0.06
clustering coefficient (simulated range, p-value)	0.59 (0.36-0.4, p=0)	0.23 (0.03-0.16, p=0)
average shortest path length (simulated range, p-value)	1.66 (1.62-1.62, p=0)	2.28 (2.51-2.81, p=1)
Degree distribution		
Average	19.1	4.7
Kurtosis	-0.7	11.4
skewness	0.3	3.3
No. of edges (%) involving the 2 main city markets	75 (15.4%)	63 (52.1%)

[1]The denominator was the number of connected nodes

2.6. AIV transmission dynamics.

Results of the sensitivity analysis of the within-market transmission model are shown in Fig. S7. The simulated prevalence of infection in markets was weakly influenced by the proportion of transmission mediated by the environment (Fig. S7, (A)). Reduction in the length of the infectious period from 48 to 24 hours resulted in an increase in the daily number of effective contacts required for achieving a 10-fold increase in the prevalence of infection. In contrast, increasing the length of the infectious period (Fig. S7, (B)) had a limited impact, as the proportion of chickens remaining in markets ≥2 days was very low. As the farm-level prevalence (i.e. the prevalence of infection in chickens daily introduced in markets) increased (Fig. S7, (C)), the daily number of effective contacts required for achieving a 10-fold increase in the prevalence of infection increased as well. Finally, the longer the sampling period over which the prevalence of infection was computed (Fig. S7, (D)), the higher the prevalence. Indeed, the proportion of infectious chickens increased between two successive introduction of chickens in the market. However, even for long durations of the sampling periods, the value of β required for the viral prevalence to increase 10-fold remained high.

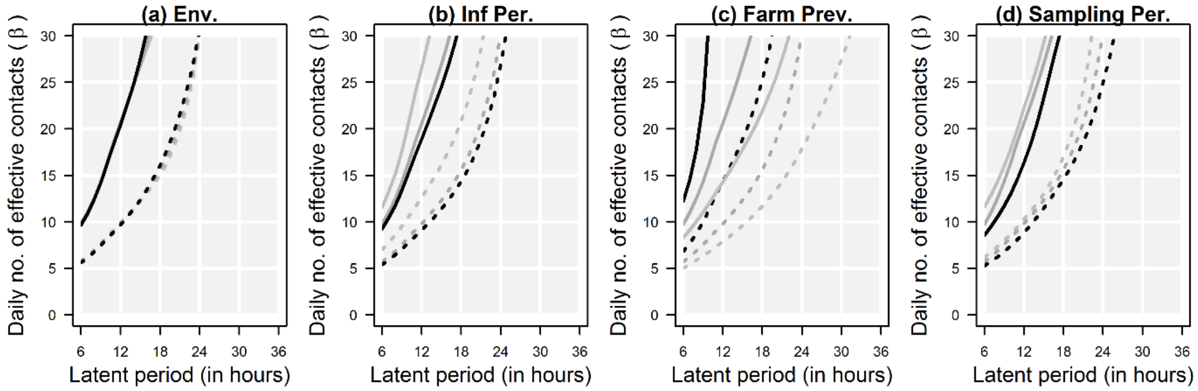

Fig. S7: Sensitivity analysis of the within-market transmission model. Values of β required for a 10-fold increase in the prevalence of infection are shown as a function of the latent period for Dhaka (solid) and Chattogram (dotted); (a) $\zeta = 0$ (light grey), 0.5 (dark grey) and 0.99 (black); (b): $T_I = 24$ (light grey), 48 (dark grey), 72 (black) hours; (c): $P_{intr} = 0.001$ (light grey), 0.005 (dark grey), 0.01 (black); (d): $T_P = 6$ (light grey), 12 (dark grey), 18 (black) hours.

3. References

1. Fournie G, *et al.* (2016) Investigating poultry trade patterns to guide avian influenza surveillance and control: a case study in Vietnam. *Sci Rep* 6:29463.
2. Moyen N, *et al.* (2018) A large-scale study of a poultry trading network in Bangladesh: implications for control and surveillance of avian influenza viruses. *BMC Veterinary Research* 14(12).
3. Croissant Y & Millo G (2018) *Panel Data Econometrics with R*.
4. Shortridge KF, *et al.* (1998) Characterization of avian H5N1 influenza viruses from poultry in Hong Kong. *Virology* 252(2):331-342.
5. Gupta SD, Hoque MA, Fournié G, & Henning J (2020) Patterns of Avian Influenza A (H5) and A (H9) virus infection in backyard, commercial broiler and layer chicken farms in Bangladesh. *Transbound Emerg Dis*.
